# Investigating the Synergistic Effects of Carvacrol and Citral-Edible Polysaccharide-Based Nanoemulgels on Shelf Life Extension of Chalkidiki Green Table Olives

**DOI:** 10.3390/gels10110722

**Published:** 2024-11-08

**Authors:** Konstantinos Zaharioudakis, Constantinos E. Salmas, Nikolaos D. Andritsos, Areti A. Leontiou, Dimitrios Moschovas, Andreas Karydis-Messinis, Eleni Triantafyllou, Apostolos Avgeropoulos, Nikolaos E. Zafeiropoulos, Charalampos Proestos, Aris E. Giannakas

**Affiliations:** 1 Department of Food Science and Technology, University of Patras, 30100 Agrinio, Greece; zacharioudakis.k@upatras.gr (K.Z.); nandrits@upatras.gr (N.D.A.); aleontiu@upatras.gr (A.A.L.); 2 Department of Material Science and Engineering, University of Ioannina, 45110 Ioannina, Greece; dmoschov@uoi.gr (D.M.); a.karyd@uoi.gr (A.K.-M.); triantafyllou.eleni@uoi.gr (E.T.); aavger@uoi.gr (A.A.); nzafirop@uoi.gr (N.E.Z.); 3 Laboratory of Food Chemistry, Department of Chemistry, National and Kapodistrian University of Athens, Zografou, 15771 Athens, Greece; harpro@chem.uoa.gr

**Keywords:** carvacol, citral, edible nanoemulgels, polysaccharides, xanthan gum, kappa-carrageenan, green table olives, shelf life

## Abstract

Modern bioeconomy and sustainability demands lead food technology in the development of novel biobased edible food preservatives. Herein, the development and characterization of novel polysaccharide (xanthan gum and kappa-carrageenan)-based nanoemulgels (NGs) enhanced with essential oil derivatives; pure citral (CT); pure carvacrol (CV); and various CT:CV ratios (25:75, 50:50, and 75:25) are presented. The obtained NGs are applied as active edible coatings for extending the shelf life of Protected Designation of Origin (PDO) green table olives of Chalkidiki. The zeta potential demonstrated the high stability of the treatments, while light scattering measurement and scanning electron microscopy images confirmed the <100 nm droplet size. EC_50_ indicated high antioxidant activity for all the tested samples. The fractional inhibitory concentration (FIC) confirmed the synergistic effect of NG with a CT:CV ratio at 50:50 against *Staphylococcus aureus* and at CT:CV ratios 25:75 and 75:25 against *E. coli* O157:H7. NG coatings with CT:CV ratios at 50:50 and at 25:75 effectively controlled the weight loss at 0.5%, maintained stable pH levels, and preserved the visual quality of green olives on day 21. The synergistic effect between CT and CV was confirmed as they reduced the spoilage microorganisms of yeasts and molds by 2-log [CFU/g] compared to the control and almost 1 log [CFU/g] difference from pure CT and CV-based NGs without affecting the growth of beneficial lactic acid bacteria crucial for fermentation. NGs with CT:CV ratios at 50:50 and at 25:75 demonstrated superior effectiveness in preventing discoloration and maintaining the main sensory attributes. Overall, shelf life extension was achieved in 21 compared to only 7 of the uncoated ones. Finally, this study demonstrates the potential of polysaccharide-based NGs in mixtures of CT and CV for the shelf life extension of fermented food products.

## 1. Introduction

Consumer demand in the modern world leans towards products with natural preservatives. Thus, food science and technology researchers have shown a sharp increase in interest in the development and investigation of natural compounds’ properties [1,2]. Simultaneously, the increasing focus on sustainability and the mindset of the circular economy are leading to the transition from synthetic food additives to the use of natural compounds with antioxidant and antimicrobial effects to tackle microbiological growth and inhibit enzymatic reactions in food [3,4,5,6,7].

Essential oils (EOs) and their derivatives have extensively been investigated due to their antioxidant and antimicrobial properties, along with their claim to be Generally Regarded as Safe (GRAS). The EO market is estimated at almost USD 24 billion and is projected to grow at an annual rate of 7.6% until 2027 [8]. EOs and their derivatives have been analyzed for their chemical composition [9]. With their ability to delay oxidative reactions or inhibit the formation of free radicals and microbial growth being well documented, their potential use as alternatives to chemical preservatives is suggested [10,11,12]. EOs and their derivatives are known for their volatility, and they are produced by a variety of plants [13,14,15,16]. These oils are primarily composed of compounds such as terpenes, phenylpropanoids, aldehydes, esters, alcohols, and ketones [17,18]. Due to their diverse chemical composition, EOs and their derivatives do not have a single defined mechanism of action against microbial cells. One commonly observed effect is cell membrane disruption, affecting proton pumps and leading to ATP depletion [19,20].

Monoterpenoid derivatives and phenylpropenes derived from EOs, such as citral (CT) (C_10_H_16_O) and carvacrol (CV) (C_10_H_14_O), are recognized for their potent bioactive properties [21,22]. Each of these compounds has well-documented antioxidant and antimicrobial properties, as supported by numerous studies [22,23]. CT, a compound formed by a mixture of neral and geranial isomers, is a linear monoterpene aldehyde that constitutes more than 85% of lemongrass EOs. This compound is primarily sourced from citrus fruits such as limes and lemons. Recognized for its citrus-based flavor, CT has been widely used in various consumer goods. The FDA has designated CT as Generally Recognized as Safe (GRAS), highlighting its extensive application in the industry [24]. CV is primarily sourced from oregano and thyme. During its metabolic pathway, hydroxy carvacrols are produced, which can target bacterial cell membranes and ATPase functions. Additionally, these hydroxy carvacrols may contribute hydrogen atoms to stabilize free radicals [25].

However, the direct application of EOs and their derivatives in foods is not suggested due to their potential to adversely affect flavors, aromas, and color, thereby deteriorating the sensory characteristics and making food products undesirable in certain contexts [26]. Additionally, the high concentration of bioactive compounds in EOs increases the likelihood and severity of toxicity or allergic reactions, necessitating strict control over their use to ensure consumer safety [27]. To address these challenges, alternative methods such as nanoencapsulation have been developed, leading to the synthesis of essential oil derivative nanoformulations [22,26,28,29,30].

Nanoencapsulation of bioactive compounds produced by self-assembly of food-derived proteins and polysaccharides provides the possibility to create nanostructures that have various characteristics, such as biodegradability, biocompatibility, effectively controlled release, and site-specific delivery, combining the advantages of both biopolymers [31,32]. Such nanostructures can improve certain sensory characteristics, such as masking unpleasant flavors. They can also enhance the bioaccessibility and bioavailability of substances, prevent oxidative reactions that can degrade their bioactivity, and regulate the release of bioactive agents. Additionally, biopolymer-based nanoemulgels (NGs) can improve the quality, stability, and bioactivity of substances by taking advantage of their reduced size at the nanoscale [33,34].

Biopolymer-based NGs are considered highly desirable nanocarriers due to their high water stability, biocompatibility, biodegradability, well-defined structure, and versatile applications in the fields of food, pharmaceuticals, and biomedicine [35]. These NGs have the potential to gradually and specifically release the encapsulated compound [36]. NGs provide some unique benefits among the available nano-delivery systems, including the higher loading capacity of bioactive compounds because of their property to form a 3D matrix [37,38]. For food industry applications, they can be produced from GRAS materials, and therefore, they have the potential to be secure, non-immunogenic, and fully/semi-biodegradable. NGs allow the grouping of several bioactive ingredients in the same formula. They are capable of encapsulating both hydrophobic and hydrophilic compounds [36,37,39].

Shen et al. [40] developed a double-network emulsion gel containing probiotics using a food-grade complex of whey protein concentrate, xanthan gum, and kappa-carrageenan. The gel’s stability and gel-like properties were enhanced by the presence of xanthan gum and kappa-carrageenan. The encapsulated probiotics showed improved viability during storage and maintained their effectiveness in balancing intestinal flora and inhibiting harmful bacteria. This suggests the potential of using food-grade biopolymers and nonthermal processing methods for functional food delivery systems. Avallone et al. [41] developed a gelling formulation using xanthan gum and kappa-carrageenan to replace animal gelatin. The combination of these two hydrocolloids offered a more stable gel network with enhanced viscoelastic properties. It was observed that the synergistic effect between xanthan gum and kappa-carrageenan significantly increased the gel strength, with the complex modulus (∣G*∣) improving as the xanthan gum concentration increased. The researchers explained that this synergy enhances the formation of double helices in the kappa-carrageenan gel network, leading to a stronger gel. Xanthan gum reduces the number of accessible water molecules, promoting easier self-association of kappa-carrageenan helices and the formation of junction zones, which contribute to improved mechanical properties. The gelation temperature remained relatively constant, ensuring the gelation process was not hindered. This combination showed promise as a green alternative to gelatin, providing strong, stable hydrogels suitable for various applications, including 3D food printing.

Xanthan gum (XG) is a high molecular weight, anionic exopolysaccharide that is produced through the aerobic fermentation of sugars by the bacterium *Xanthomonas campestris* [42]. Structurally, it features a β-(1 → 4) linked glucose backbone with charged trisaccharide sidechains. Known for being non-toxic, hydrophilic, and biodegradable, XG is extensively utilized in the food, pharmaceutical, and cosmetic sectors due to its outstanding rheological properties. It dissolves readily in both cold and hot water, hydrates swiftly, and generates high viscosity even at low concentrations [43]. In the pharmaceutical industry, XG is employed as a controlled release agent in solid dosage forms and functions as a thickening, suspending, and stabilizing agent in liquid formulations [44].

Kappa-carrageenan (KC) is an anionic polysaccharide extracted from red edible seaweeds, specifically from Gigartinales. It is composed of alternating 1,3-linked galactose 4-sulfate and 1,4-linked 3,6-anhydro-D-galactose residues. κ-carrageenan is most sensitive to potassium (K+) ions, which enable it to form rigid, thermally reversible aqueous gels [45,46,47]. Known for its high gel-forming ability and thermoreversible gelation properties, κ-carrageenan forms strong, stable gels upon cooling and melts when reheated [41]. It is widely used in the food industry to improve texture, stability, and viscosity in products like dairy, meat, and plant-based foods. Additionally, κ-carrageenan’s interactions with proteins enhance the rheological properties of food systems, making it valuable in creating desirable textures and structures in various applications [47].

Chalkidiki green table olives constitute a traditional product registered in the Commission Implementing Regulation (EU) no. 426/2012 as a Protected Destination of Origin (PDO) product [48]. The total production of such a finished Chalkidiki table olive product exceeds 145,000 tons. Notwithstanding the fact that olive oil is a common practice to preserve table olives, according to Eurostat [49], in January 2024, the olive oil price surged by 50% higher than in January 2023 due to depreciation, making it a not-so-beneficial option. Various studies have focused on flavoring olive oil with herbs and essential oils, or oleogels demonstrating significant shelf life extension and consumer acceptance. However, there is limited research available to investigate the shelf life of table olives with polysaccharide-based NGs enhanced with essential oils.

To the best of our knowledge, the innovative points of the current study are (i) the development and characterization of such polysaccharide-based NGs using xanthan gum and kappa-carrageenan, enhanced with derivatives of essential oils, (ii) the application of such NGs as edible active coatings for extending the shelf life of Chalkidiki traditional green PDO table olives, and (iii) the examination of potential synergistic effects of CV and CT nanoencapsulated in such novel NGs.

## 2. Results and Discussion

### 2.1. Particle Size Z Potential Dynamic Light Scattering (DLS) and Scanning Electron Microscopy (SEM) Image Characterizations of NG Coatings

The determined zeta potential values of all the obtained NG coatings are shown in Table 1.

The results from the data in the table demonstrate that the coatings are stable and with high zeta potential values ranging between −35.28 and −45.72 mV. The most noticeable difference is that the zeta potential for individual derivative oil NGs demonstrate higher values, although the NG mixtures do not have statistically significant lower zeta potential at the 5% level. The anionic character of xanthan gum and kappa-carrageenan, combined with the negative ions on the surface of the oil droplets, explains the negative charge of the NGs [50]. Moreover, the presence of functional groups as found in essential oils, such as hydroxyl (–OH) and carboxyl (C=O) groups, seems to affect the zeta potential [51]. It is suggested that the hydroxyl groups of CV form stronger hydrogen bonds and electrostatic interactions with the NG surface, resulting in more negative zeta potential values. On the other hand, the zeta potential value of CT NG is lower than that of the CV NG zeta potential value, implying a weaker interaction between the CT molecule and polysaccharide matrix. It is suggested that CV’s higher polarity and potential for stronger electrostatic repulsion and ion adsorption contribute to these variations.

The zeta potential values reported here are consistent with those observed in recent studies on EO-based NGs, which highlight similar trends in stability and charge characteristics [52,53,54,55,56,57].

**Figure 1:**
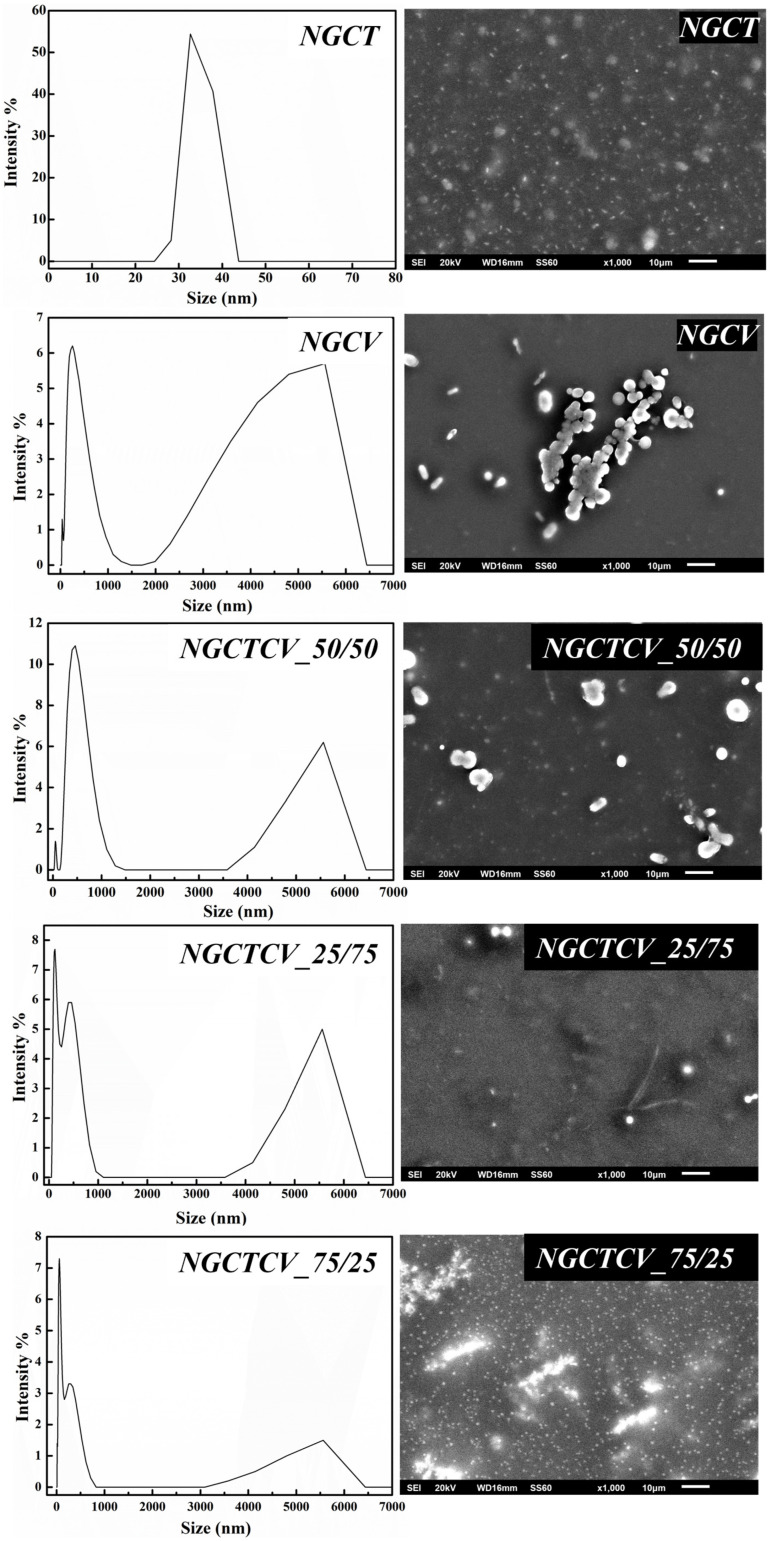
Dynamic light scattering data and SEM images of the materials NGCT, NGCV, NGCTCV_50/50, NGCTCV_25/75, and NGCTCV_75/25.

In Figure 1, the DLS recorded data (in the left part) and the SEM images for all the obtained NGs are shown.

As shown in Figure 1 (left part), the NGCT sample particle size averages ~34 nm (~100%). The NGCV sample showed an average particle size of ~310 nm (~70%) and of 4300 nm (~24%). The DLS data of the samples NGCTCV_50/50, NGCTCV_25/75, and NGCTCV_75/25 show a major population of the particles between 140 nm and 480 nm and the rest in the range of μm. As it is observed in the right part of Figure 1, SEM images (see the right part in Figure 1) agree with the data provided by DLS measurements, and they confirm the distribution and morphology of NGs.

### 2.2. Antioxidant Activity EC_50_

Table 2 enlists the average EC_50_ values derived from the DPPH assay for the examined NG treatments.

From the EC_50_ values listed in Table 2 for all the obtained NGs, it demonstrated that NGCV exhibited the highest free radical antioxidant activity and NGCT the lowest one. All NGs containing mixtures of CV and CT exhibited lower antioxidant activity than the NGCV and did not indicate any synergistic effect. In all the NGCTCV samples, it is obtained that the higher the CV concentration, the higher the obtained antioxidant activity or the lower the CT concentration, the higher the obtained antioxidant activity. The results presented here are similar to that presented by Al-Mansori et al. [58], which claimed that no synergistic antioxidant activity of thymol and CV mixture was observed. From the results listed in Table 2, it is also demonstrated that the obtained nanodroplet size of the NGs affects the recorded antioxidant activities. It looks like the smaller the obtained NG nanodroplet size, the higher the antioxidant activity. This result is in accordance with previous ones, where it is claimed that the nanodroplets of nanoemulsions can increase the ability of lipophilic compounds such as essential oils to be biologically active by increasing the surface area per unit of mass [59,60].

Moreover, various studies are in accordance with these results, showing a higher efficacy of CV formulations against CT ones [61,62,63,64,65,66].

### 2.3. Antibacterial Activity of NGs

#### 2.3.1. Agar Diffusion Zone

In Table 3 is the obtained antibacterial activity of the examined NG samples against two bacterial strains, *Escherichia coli* and *Staphylococcus aureus*, measured by the agar diffusion method. The measured zones of inhibition, expressed in millimeters (mm), indicate the effectiveness of each treatment. The findings, expressed as the average ± standard deviation (SD), are outlined in Table 3. Figure S1 displays representative images of the observed diffusion zones for all the tested NG derivatives.

Overall, the analysis of the data provided indicated that the most important finding is that the mixtures of CT and CV in all the ratios exhibited higher antibacterial activity than the individual NGs. Among these, the 50:50 ratio (NGCTCV50:50) demonstrated the largest and most significant (*p* < 0.05) inhibition zones against both bacterial strains.

To begin with, for *S aureus*, all NG samples exhibited substantial antibacterial activity. NGCTCV50:50 exhibited the largest inhibition zone of 20.06 mm, approximately one-third larger than the 15 mm zone observed for NGCV and almost double the 11 mm zone for NGCT (*p* < 0.05). NGCTCV25:75 and NGCTCV75:25 also showed considerable activity with inhibition zones of 16.5 mm and 18.34 mm, respectively, both surpassing the individual NG NGCV and NGCT.

For *S. aureus*, the antibacterial activity was generally lower than for *E. coli*. NGCTCV50:50 again showed the largest inhibition zone of 15 mm, which was 25% larger than the 12 mm zone observed for NGCV and almost double the 9 mm zone for NGCT. Other mixture ratios, such as NGCTCV25:75 and NGCTCV75:25, had inhibition zones of 12.34 mm and 12.05 mm, respectively, still surpassing the individual NGs.

There is a noticeable difference in the antibacterial activity against the two examined strains. All NG samples exhibited higher antimicrobial activity against *E. coli* than *S. aureus*. NGCTCV50:50 has an inhibition zone of 20.06 mm for *E. coli* O157:H7, which is approximately one-third larger than the 15 mm zone for *S. aureus*. Similarly, the NGCV exhibited a 15 mm inhibition zone for *E. coli* O157:H7, which is 25% larger than the 12 mm zone for *S. aureus*. NGCT showed an 11 mm zone for *E. coli*, which is over 20% larger than the 9 mm zone for *S. aureus*.

Among the various ratios of CT and CV tested, only the 50:50 ratio exhibited the most pronounced antibacterial activity against both bacterial strains. This observation indicates a synergistic effect occurs as a higher effect than the individual NGs.

Various studies have claimed that there is a synergistic effect of CT and CV against several bacteria such as *L. monocytogenes*, *L. innocua* [67], and *C. sakazakii* [68]. According to the study conducted by Silva-Angulo et al. [67], CT and CV may have synergistic effects due to their targeting of different sites in bacterial cells. It has been suggested by the researchers that CV increases the outer membrane permeability, allowing CT to enter the cytoplasm and interact with proteins and nucleic acids. Combining these substances could improve CT’s and CV’s solubility and enhance their antibacterial effect [69]. Further investigation is needed to understand the molecular action of individual or combined treatments with natural substances.

Therefore, the 50:50 ratio demonstrates the optimal combination of CT and CV, as it maximizes the benefits of both antibacterial agents. Overall, the synergistic effect of CV and CT is, for the first time, reported in such xanthan-carrageenan-based NGs.

#### 2.3.2. MIC, MBC, and FIC

Table 4 provides the MIC, MBC, and FIC results for the examined NG samples against *Escherichia coli* O157:H7 and *Staphylococcus aureus*. The MIC and MBC values reported in μg/mL indicate the lowest concentration of NG required to inhibit the growth of each bacterial strain, and the FIC values indicate the effectiveness of each derivative, as well as the synergistic effects of combined treatments.

The most essential finding from Table 4 is that the mixtures of CT and CV exhibited significantly enhanced antibacterial activity due to their synergy or additive effect compared to individual derivative compound NGs. Specifically, the NGCTCV25:75 and NGCTCV75:25 mixtures demonstrated the highest synergistic effects against *E. coli O157:H7*, with MIC and MBC values of <125 μg and FIC of 0.375. Similarly, the NGCTCV50:50 mixture demonstrated the highest antimicrobial activity against *S. aureus*, with MIC and MBC values of <125 μg and FIC of 0.375, indicating a synergistic effect. This highlights that specific ratios of CT and CV not only enhance antibacterial efficacy but also work synergistically to inhibit and or kill bacterial strains more effectively than the individual components alone.

Regarding *E. coli* O157:H7, the MIC and MBC values indicate that NGCTCV25:75 and NGCTCV75:25 were the most effective, with both achieving inhibition and bactericidal action at concentrations of <125 μg. The 50:50 mixture (NGCTCV50:50) showed MIC and MBC values of <250 μg, which was still more effective than the individual NGs NGCT and NGCV, with MIC and MBC values of <1000 μg and <500 μg, respectively. The FIC values for these combinations highlight the significant synergy (FIC < 0.5) for NGCTCV25:75 and NGCTCV75:25, with a FIC value of 0.375, indicating a strong synergistic effect. The NGCTCV50:50 mixture showed an additive effect (FIC < 1.0) with a FIC value of 0.75.

NGCTCV50:50 exhibited the most potent antibacterial activity with MIC and MBC values of <125 μg, significantly lower than the individual NG NGCT and NGCV, which had MIC and MBC values of <1000 μg and <500 μg, respectively. NGCTCV25:75 and NGCTCV75:25 had MIC and MBC values of <250 μg, showing notable efficacy. The FIC values for these combinations showed significant synergy for NGCTCV50:50, with a FIC of 0.375, and additive effects for NGCTCV25:75 and NGCTCV75:25, with FIC values of 0.75.

Comparing the two bacterial strains, *E. coli* O157:H7 exhibited greater susceptibility to the NG treatments than *S. aureus*. The MIC and MBC values for *E. coli* were consistently lower, indicating higher effectiveness at lower concentrations. For instance, the MIC and MBC values for NGCTCV25:75 and NGCTCV75:25 were <125 μg for *E. coli* compared to <250 μg for *S. aureus*. The FIC values also highlight stronger synergistic effects against *E. coli* (FIC of 0.375) compared to the additive effects against *S. aureus* (FIC of 0.75). These findings emphasize that the combinations of CT and CV, particularly in certain ratios, provide enhanced antibacterial activity and synergy, especially against *E. coli* O157.

Findings similar to ours have been found for the additive effect of CT and CV against *E. coli* [70]. Kim et al. [71] found that CV and CT can control *Listeria* growth at 25% of their MICs. Zanini et al. [72] suggested CT and CV could enhance antibacterial activity. Synergistic effects of cinnamaldehyde and thymol, CV and eugenol, and thymol and eugenol have also been reported [73]. Cao et al. [68] reported the FICI of the synergistic combination is 0.5, with CT and CV at a concentration of 1/4 MIC.

#### 2.3.3. Time-Killing Assay

The time-killing assay was used to confirm the synergistic effect of CT and CV on *Staphylococcus aureus* and *Escherichia coli*. This method provided detailed insights into the antibacterial efficacy of various NG samples over a 24-h period, measured in log10 CFU/mL.

**Figure 2:**
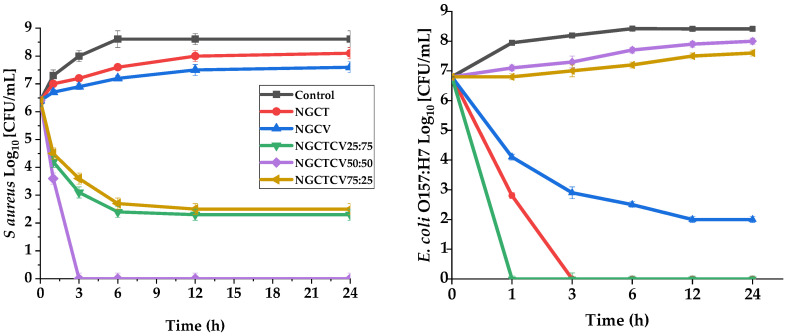
Time-killing assay of NGs against *S aureus* (**left**) and *E coli* (**right**).

The most noticeable observation in Figure 2 is the superior efficacy of the NGCTCV 50:50 mixture, which completely eradicated *E. coli* by the 3rd hour and maintained this level up to the 24th hour. Additionally, for *S. aureus*, this mixture reduced the bacterial count to 0 log10 CFU/mL by the 3rd hour and sustained this reduction throughout the testing period. This indicates a remarkable synergistic effect when CT and CV are combined in equal proportions.

Analyzing the NGs, NGCTCV 50:50 showed a 2-log reduction (*p* < 0.05) in *S. aureus* within the first hour, leading to a total population loss. For *E. coli*, the NGCTCV 75:25 mixture also resulted in a complete eradication (*p* < 0.05) by the first hour, demonstrating its strong antibacterial activity. Comparatively, NGCT and NGCV were less effective, with NGCT only reducing *S. aureus* by about 0.5 log and NGCV achieving a similar reduction for both bacteria. The NGCTCV 25:75 mixture achieved a significant (*p* < 0.05) 4.1 log reduction for *E. coli* within the first hour and maintained a steady low count, indicating its potent antibacterial effect.

Comparing the NGs’ performance in both bacteria, the mixtures generally exhibited stronger antibacterial effects against *E. coli* than *S. aureus*. NGCTCV 50:50 eradicated *E. coli* by the 3rd hour, while achieving the complete reduction of *S. aureus* took slightly longer. The NGCTCV 75:25 mixture demonstrated complete eradication of *E. coli* by the 1st hour, whereas, for S. aureus, the same mixture only reduced the population to about 2.5 log10 CFU/mL (*p* < 0.05) by the 24th hour. This indicates that *E. coli* O157:H7 is more susceptible to the synergistic effects of CT and CV than *S. aureus*.

The results highlight the enhanced efficacy of mixture derivative compound NGs against the individual components. The synergistic effect of CT and CV, particularly in the 50:50 and 75:25 ratios, significantly improves antibacterial activity, leading to the complete eradication or substantial reduction of bacterial populations. This indicates the potential of these combined NGs as powerful antibacterial agents, offering greater effectiveness than individual CT or CV NGs.

Silva-Angulo et al. [67] found that CV and CT combined at 25% MIC effectively inhibited *Listeria* growth. Similarly, the combination at 1/4 MIC completely inhibited *C. sakazakii* growth after 8 h and, at 1/2 MIC, completely killed the cells after 1 h. These results confirm that CV and CT have a synergistic inhibitory effect on *C. sakazakii* [68].

### 2.4. Application of Obtained NGs in Olives Preservation

#### 2.4.1. Yeasts and Mold and LAB Bacteria LOG (CFU/gr^−1^)

**Figure 3:**
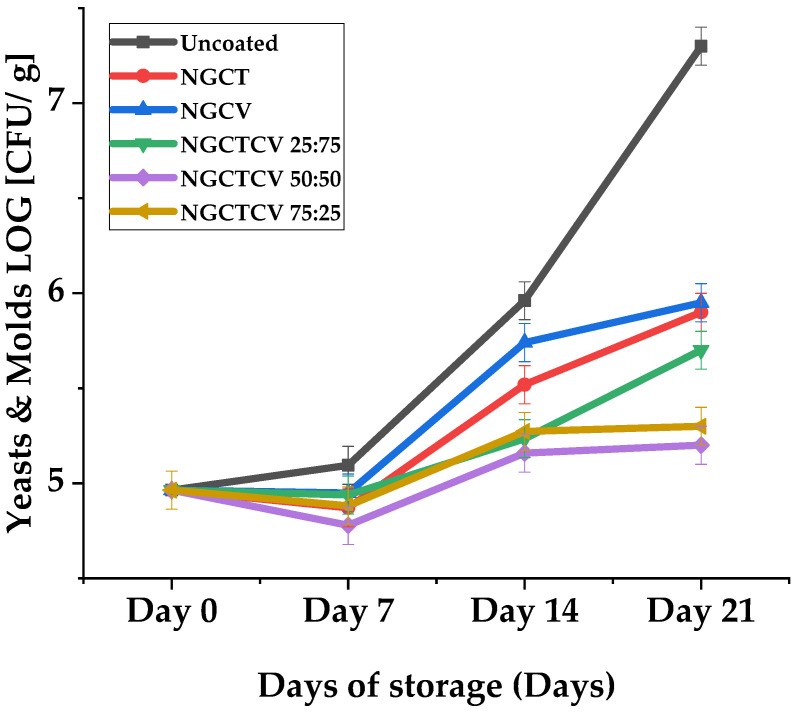
Evaluation of yeast and mold bacteria populations on olives during the 21-day storage period.

Figure 3 illustrates the evaluation of yeast and mold populations on olives during the 21-day storage period, which was conducted to confirm the synergistic effect of CT and CV in preserving Chalkidiki green table olives.

Among the nanogel treatments, NGCTCV 50:50 exhibited the strongest reduction in yeast and mold populations throughout the 21-day storage period, consistently outperforming both individual components and other mixtures.

In particular, on Day 21, the yeast population in olives treated with NGCTCV 50:50 was 5.86 log [CFU/g], significantly lower than the 7.19 log [CFU/g] observed in the control sample, indicating a strong preservative effect.

NGCTCV 50:50 exhibited a yeast population reduction of approximately 2-log [CFU/g] compared to the control by Day 21. This represents a nearly two-fold reduction in yeast population. Similarly, both NGCTCV 25:75 and NGCTCV 75:25 showed substantial reductions, with yeast populations of slightly above 5 log [CFU/g], respectively, on Day 21, indicating synergistic preservation but slightly less so than the 50:50 mixture. In contrast, the individual NG NGCT and NGCV showed less effectiveness, with yeast populations of 5.93 and 5.80 log [CFU/g], respectively, by Day 21.

Comparing the NGs’ performance over the storage period, the combined NGs consistently outperformed the individual component NGCT and NGCV. Specifically, on Day 14, the NGCTCV 50:50 mixture had a yeast population of 5.16 log [CFU/g] compared to 5.52 for NGCT and 5.74 for NGCV. This pattern continued through Day 21, where the NGCTCV 50:50 mixture maintained the lowest yeast population among all the treatments.

The results from pairwise comparisons further reinforced these findings. For example, on Day 7, the yeast population for NGCTCV 50:50 was significantly lower than NGCV, NGCTCV 25:75, and the control (*p* < 0.05). By Day 14, the NGCTCV 50:50 mixture continued to show significant reductions compared to NGCV and the control, with *p*-values of 0.039 and <0.001, respectively. On Day 21, the comparison between NGCV and NGCTCV 50:50 revealed a significant difference (*p* < 0.05) (see Table S5).

These results indicate that the mixture derivative compound NGs, particularly the NGCTCV 50:50 ratio, provide enhanced preservative effects against yeast compared to individual CT or CV NGs. The synergistic effect of these mixtures not only effectively reduces yeast populations but also maintains this reduction consistently over the storage period, demonstrating their potential as powerful preservative agents for olives.

Mold growth during storage can lead to the appearance of visible mycelia. These microorganisms are generally recognized as spoilage agents, contributing to undesirable changes, such as softening of the flesh and the development of off-flavors, moldy taste, and altered appearance. In table olives, the most commonly identified mold genera are Aspergillus and Penicillium [74]. CT and CV are well documented for their antibacterial and antifungal activity [75,76]

**Figure 4:**
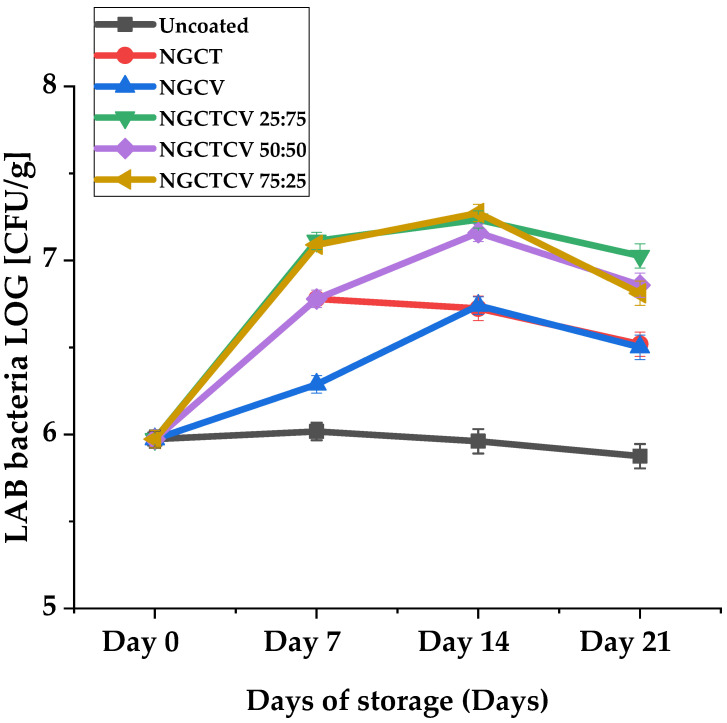
LAB growth of olives during 21-day storage period.

In Figure 4, the LAB growth mean values of olives during the 21-day storage period are plotted. The evaluation of the LAB bacteria population on olives during the 21-day storage period reveals some statistically significant trends that highlight the effects of different NG treatments. The most noticeable difference was that certain mixtures of CT and CV significantly increased the LAB population on olives, contrary to the expected antibacterial activity.

On day 7, the control sample had a LAB population of 6.0 log [CFU/g], whereas the NGCTCV 25:75 and NGCTCV 75:25 mixtures showed higher populations of 7.1 log–log [CFU/g], which was almost two-fold higher than the control. The NGCTCV 50:50 mixture also had a LAB population of 6.8 log–log [CFU/g], significantly higher than the control, demonstrating a similar trend. These differences were statistically significant at the 5% level (*p* < 0.05), indicating a clear increase in LAB population due to the NG treatments (see Table S6).

By Day 14, the control’s LAB population decreased to 5.5 log–log [CFU/g], while the NGCTCV mixtures continued to show higher LAB counts. NGCTCV 25:75 and NGCTCV 50:50 both reached 7.2 log–log [CFU/g], and NGCTCV 75:25 reached 7.3 log–log [CFU/g], indicating more than a two-fold increase compared to the control. These differences were also statistically significant at the 5% level, reinforcing the observation that the NG mixtures sustain higher LAB populations over time.

By Day 21, the LAB population in the control sample further decreased to 5.1 log10 CFU/mL. Notwithstanding, the NGCTCV mixtures maintained higher populations: NGCTCV 25:75 had 7.0 log [CFU/g], NGCTCV 50:50 had 6.9 log10 log [CFU/g], and NGCTCV 75:25 had 6.8 log10 log [CFU/g]. This represents a nearly two-fold increase compared to the control. The observed differences were statistically significant at the 5% level, confirming the sustained higher LAB populations in the presence of NG mixtures.

These statistically significant trends suggest that, while NG mixtures may control the growth of yeast and mold populations, they do not seem to affect the fermentation process of LAB bacteria differently than expected, indicating potential interactions that warrant further investigation.

The breakdown of polysaccharides by lactic acid bacteria relies on different hydrolases. In fermented foods, this polysaccharide degradation supplies energy to the lactic acid bacteria and produces various beneficial substances for humans [77]. Papapostolou et al. [78] reported that, while the lactic acid bacteria populations on green table-fermented olives were not affected by the use of essential oils during storage, the yeast and mold populations experienced a more controlled increase compared to the control sample. These results indicate the potential use of probiotic microorganisms that could be used in the future.

#### 2.4.2. Weight Loss Analysis

Table 5 represents the results from the percentage weight loss analysis of Chalkidiki green table olives over a storage period of 21 days.

The most noticeable observation is that NGs effectively managed to control the weight loss of olives over the 21-day storage period.

The results on Day 7 indicate that all the NG treatments significantly reduced weight loss compared to the uncoated olives. NGCT and the mixtures exhibited the lowest reductions, similar to NGCTCV 50:50. Statistically, significant differences were noted between the uncoated olives and those coated with NGCV (*p* = 0.012), NGCT (*p* = 0.037), and NGCTCV 75:25 (*p* = 0.037), indicating more effective treatments.

By Day 14, among the NG coatings, NGCT and NGCTCV at ratios 25:75 and 75:25 demonstrated the most effective control, with the mixtures generally performing like the individual essential oil coatings. Statistically significant differences were observed when comparing the uncoated olives to those coated with NGCTCV 25:75 (*p* = 0.010), NGCTCV 50:50 (*p* = 0.012), and NGCTCV 75:25 (*p* = 0.010).

Finally, on Day 21, the observed trend continued, with the NG formulations NGCTCV at ratios 25:75 and 50:50 to demonstrate the most effective control overweight loss. Although the individual essential oil NGs exhibited considerable efficacy, the NG mixtures, particularly NGCTCV 25:75, outperformed them. Statistically significant differences were identified between the uncoated olives and those treated with NGCV (*p* = 0.045), NGCTCV 25:75 (*p* = 0.007), NGCTCV 50:50 (*p* = 0.009), and NGCTCV 75:25 (*p* = 0.011) but also between NGCTCV 25:75 and NGCV (*p* = 0.045).

**Figure 5:**
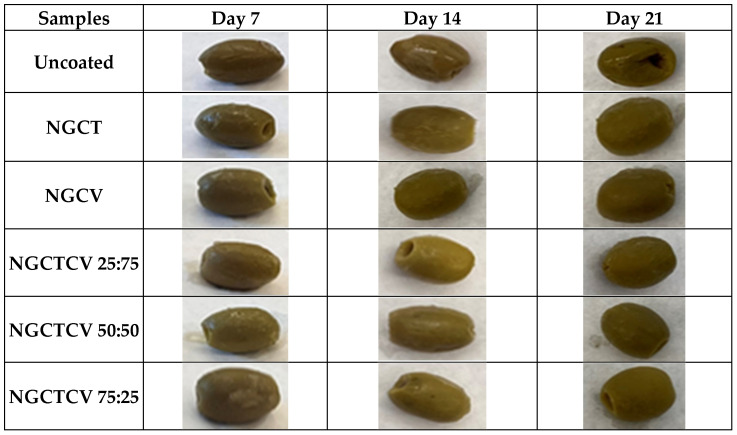
Visible effect of NGs on moisture control compared to the uncoated sample on Days 7, 14, and 21.

Figure 5 visualizes the effect of novel essential oil NG coatings applied on Days 7, 14, and 21. The mixture of CT and CV exhibited the highest efficacy compared to the individual components, particularly at ratios of NGCTCV 50:50 and 25:75. It is evident that the Chalkidiki green table olives coated with these NGs showed no skin cracks, and their color remained unchanged throughout the examined period. However, olives treated with CV NG experienced skin shrinkage, and their color darkened as early as Day 14.

These findings underscore the superior efficacy of the NG coatings, especially the CT–CV mixtures, in managing weight loss in green table olives over the 21-day period. The findings suggest that these NGs are valuable physical preservatives and viable alternatives to synthetic ones. Their ability to maintain the product’s freshness and quality makes them a promising commercial product. According to Priya et al. [79], polysaccharide coatings may prevent the loss of volatile compounds present in food products, maintain fruit firmness, and reduce the weight loss to some extent. Moisture loss can be minimized by the application of coating, which acts as a barrier. To prevent the deterioration of fruits and vegetables, a coating must provide at least a minimal level of permeability barrier against water vapor, since they lose freshness upon the loss of water. Ali et al. [80] reported in their study on the application of carboxymethyl cellulose coatings on mango fruits that they managed to control the weight loss, as well as maintain color and firmness. Similarly, Kerdchoechuen et al. [81] also found that starch-based edible coatings on minimally processed pummelo effectively manage weight loss during storage, with cassava starch showing superior results in preserving the fruit’s quality and appearance.

Martillanes et al. [82], in their study, found that incorporating natural antioxidants, such as polyphenols and essential oils, into absorbent pads effectively controls moisture, prevents spoilage, and enhances the safety of food products.

Finally, the findings suggest that these NGs are valuable physical preservatives and viable alternatives to synthetic ones. Their ability to maintain the product’s freshness and quality makes them a promising commercial product.

#### 2.4.3. pH Analysis

Table 6 represents the results from the pH analysis of Chalkidiki green table olives over a storage period of 21 days.

The most noticeable observation is that olives coated with NGs during the first 7 days of the storage period exhibited a significant fall in pH values. The most dramatic pH decreases were observed in the mixtures NGCTCV 50:50 and NGCTCV 25:75, which had pH values of 3.77 and 3.79, respectively. Statistically significant differences were noted between uncoated olives and those coated with NGCTCV 50:50 (*p* = 0.037), NGCTCV 25:75 (*p* = 0.005), and NGCT (*p* = 0.005). Furthermore, by Day 14, the pH levels of all the samples began to increase, with uncoated olives showing the highest pH rise to 4.65. Among the NG coatings, NGCTCV 75:25 and NGCT continued to demonstrate lower values than those of the uncoated olives. Statistically significant differences were observed between the uncoated olives and those coated with NGCTCV 50:50 (*p* = 0.005), NGCTCV 25:75 (*p* = 0.037), and NGCTCV 75:25 (*p* = 0.037).

Moreover, on Day 21, the trend continued, with the NG formulations NGCTCV 50:50 and NGCTCV 75:25 demonstrating the most effective control over the pH levels. Although the individual essential oil NGs also showed efficacy, the NG mixtures, particularly NGCTCV 75:25, maintained a lower pH compared to the uncoated samples. Statistically significant differences were identified between the uncoated olives and those treated with NGCTCV 75:25 (*p* = 0.013), NGCTCV 50:50 (*p* = 0.034), and NGCV (*p* = 0.001), highlighting the superior performance of the NG mixtures.

The results indicate that the presence of polysaccharide edible NG coatings provided a nutrient source for lactic acid bacteria, leading to the production of acid and a subsequent lowering of the pH values. The essential oils incorporated in the NGs did not seem to tackle the growth of the bacterial population. Finally, NGs containing mixtures of essential oils NGCTCV 50:50 and NGCTCV 75:25 were most effective in controlling the pH levels over the storage period, enhancing the preservation and quality of the olives.

#### 2.4.4. Lab Colorimetry

Table 7, Table 8 and Table 9 demonstrate the results of the L*a*b* colorimetry analysis, which examined the discoloration of Chalkidiki green table olives coated with various NGs over a 21-day storage period.

To begin with, the most prominent outcome is that all NG coatings on olives, except for NGCV, effectively controlled the discoloration of olives during the 21-day storage period. Indeed, the discoloration rate was slower, and the values of the L*a*b* parameters were higher for the coated olives compared to the uncoated ones.

Regarding the L* parameter, the decrease in values, indicating the darkening of olives over time, was most pronounced in the NGCV-coated and uncoated olives. Statistically significant changes occurred between Days 7 and 21 and Days 14 and 21. The statistically significant differences observed between NGCV vs. NGCT (*p* = 0.026) and NGCV vs. NGCTCV 50:50 (*p* = 0.005) suggest that NGCV was less effective in preventing darkening compared to the other NG coatings. Similarly, the differences between NGCTCV 75:25 vs. NGCTCV 50:50 (*p* = 0.014) and uncoated vs. NGCTCV 50:50 (*p* = 0.026) indicate that the essential oil mixtures, particularly NGCTCV 50:50, provided superior protection against darkening. NGCV demonstrated L* values similar to uncoated olives. This may occur, as CV is prone to oxidation when exposed to air and light, while NGCTCV 25:75 was the most effective in maintaining higher L* values.

With respect to the a* parameter, which reflects the shift toward red and indicates the loss of the green hue in olives, statistically significant changes were observed between Days 0 and 14, particularly in the NGCTCV 25:75 (*p* = 0.005), NGCTCV 50:50 (*p* = 0.013), and NGCTCV 75:25 (*p* = 0.024) coatings. These mixtures effectively stabilized the a* values, reducing the loss of the green hue compared to the NGCV and uncoated olives. Significant differences (*p* < 0.05) appeared between pairs like NGCTCV 50:50 vs. NGCV (*p* = 0.007), NGCTCV 25:75 vs. NGCV (*p* = 0.014), and uncoated vs. NGCV (*p* = 0.013), with NGCTCV 25:75 showing the best control.

Furthermore, the b* parameter, representing the blue–yellow axis, with higher values indicating a stronger yellow hue, showed significant changes (*p* < 0.05) between Days 7 and 21 for NGCV- and NGCTCV-coated olives. NGCV exhibited a rapid decline in b* values, indicating a faster loss of the yellow hue, while NGCTCV mixtures retained the yellow color more effectively than the NGCV and uncoated samples. Significant differences (*p* < 0.05) were found between pairs such as NGCT vs. NGCV (*p* = 0.003), NGCTCV 25:75 vs. NGCTCV 50:50 (*p* = 0.042), NGCTCV 25:75 vs. uncoated (*p* = 0.009), and NGCTCV 75:25 vs. NGCTCV 50:50 (*p* = 0.047). NGCTCV 25:75 was particularly effective in maintaining higher b* values, preserving the yellow hue better than other coatings.

In conclusion, while NGCV-coated olives displayed discoloration trends similar to the uncoated samples, the essential oil mixtures in the NGCTCV coatings proved to be more effective in controlling the discoloration rate across all L*a*b* parameters. Among the mixtures, NGCTCV 25:75 consistently outperformed the others, demonstrating the best preservation of the color of Chalkidiki green table olives during the 21-day storage period. These findings highlight the superior effectiveness of essential oil mixtures in NGs over individual oils in maintaining an olive color.

#### 2.4.5. Sensory Analysis

Table 10 enlists the sensory analysis results for the NG coatings on Chalkidiki green table olives, highlighting the effects of different treatments over a period of time.

The most noticeable difference is that, while uncoated olives showed a marked decline in sensory qualities such as taste, odor, color, and texture, the olives treated with NG coatings retained significantly better sensory properties. In particular, the mixed NG coatings (NGCTCV) demonstrated the highest efficacy in preserving the olives’ overall sensory attributes throughout the examined period.

The mixed NGs, especially in ratios of NGCTCV 50:50 and NGCTCV 25:75, outperformed both the individual oil-based NG NGCV and NGCT and the uncoated olives in nearly every parameter. Specifically, they maintained superior taste, odor, color, and texture, with significantly higher scores. The results are significant at the 5% level, indicating that the use of mixed NG coatings can be an effective strategy for enhancing the shelf life and sensory appeal of table olives.

The mixed NG coatings not only retained the olives’ freshness but also prevented skin cracking and color darkening, issues commonly observed in the uncoated olives and, to a lesser extent, in those coated with individual oils. Even though individual NG coating NGCV and NGCT showed some level of improvement over the uncoated samples, they were less effective than the mixed NG coatings. For instance, olives coated with NGCV showed signs of skin shrinkage and a noticeable decline in sensory qualities, particularly after Day 7. NGCT performed slightly better than NGCV but still did not match the efficacy of the mixed formulations in terms of maintaining the desired sensory characteristics. The benefit of using the mixed NG coatings included better preservation of taste and odor, maintenance of the olives’ vibrant color, and prevention of textural degradation. However, a potential con could be the complexity of preparing the mixed formulations compared to single-component coatings. Uncoated olives, by contrast, suffered from rapid quality loss, including flavor deterioration, odor decline, and skin darkening, rendering them less desirable for consumption.

Notwithstanding the fact that, in our previous study, the essential oil nanoemulsions were proven to be an alternative solution to maintain the main sensory categories in meat [22], this is the first time reporting that the mixture of the essential oils has an extra benefit on the examined aspects of sensory analysis on fermented food products such as Chalkidiki green table olives.

Overall, stable negatively charged NGs demonstrated high effectiveness in the preservation of Chalkidiki PDO green table olives, achieving a 2-log reduction in yeast and mold growth while not affecting the LAB population growth. The NGCTCV 50:50 and 25:75 mixtures effectively controlled weight loss, maintaining the olive quality over 21 days.

The modern consumer trend towards replacing synthetic preservatives in fermented food products with natural alternatives has increased. Notwithstanding the fact that EOs constitute a promising solution due to their antimicrobial and antioxidant properties, challenges from direct application indicate the need for another technology of using them, such as nanoencapsulation. In this study, polysaccharide-based NGs enhanced with essential oil derivatives, individually and in mixtures of CT and CV, were successfully developed and evaluated for their potential as natural preservatives for the shelf life extension of fermented products such as Chalkidiki PDO green table olives.

The zeta potential results indicated a high stability of the NGs, making them an ideal option as preservative agents due to their effectiveness. All NG coatings exhibited high negative values, indicating effective colloidal stability and ensuring that the active compounds remain evenly dispersed throughout the NG matrix. Among them, the NGCV NG showed the highest zeta potential, likely due to the strong bonding and electrostatic interactions between the examined essential oil derivatives and the negatively charged polysaccharides xanthan gum and kappa-carrageenan, and our results are in accordance with various studies [12,50,52,53,54,55,56,57].

Furthermore, the NGs showed significant antioxidant activity, as examined by the DPPH method. The NGCV NG outperformed the rest of the coatings, needing the lowest quantity to achieve a 50% radical reduction. The results are in accordance with other studies [83]. However, the antioxidant activity of the NG mixtures did not demonstrate a significant synergistic effect, with the NGCTCV mixtures showing lower activity than NGCV individually, and this result is in agreement with Al-Mansori et al. [58]. One possible explanation is that the phenolic hydroxyl group constitutes the main reason for CV’s high antioxidant activity, which enables strong free radical stabilization, while CT lacks this group and terpene aldehyde is more effective in lipid oxidation, making it less effective [84,85].

The antimicrobial preliminary tests results, including MIC, MBC, FIC, and time-killing assay, demonstrated that mixtures of CT and CV exhibit enhanced antibacterial activity compared to individual NGs. In particular, the NGCTCV 50:50 mixture showed a bactericidal effect against *Escherichia coli* O157:H7 and significantly reduced *Staphylococcus aureus* populations within hours. The FIC index showed the synergistic effect of NGCTCV 25:75 and 75:25 against *E. coli* O157:H7 and the synergistic effect of NGCTCV 50:50 against *S. aureus*. The synergistic effect of CT and CV is well documented in several studies and is explained as the result of different sites targeted within bacterial cells [67]. Specifically, CV increases the permeability of the bacterial membrane, allowing CT to penetrate more effectively into the cytoplasm, improving the overall antimicrobial effect. The bactericidal effect of the synergism is well documented in various studies against pathogens like *L. monocytogenes*, *C. sakazakii*, and *E. coli* [68,69].

The application of NG coatings on Chalkidiki PDO green table olives effectively controlled the growth of yeast and molds during the 21-day storage period. Among the treatments, NGCTCV 50:50 was particularly effective in reducing yeast populations by 2-log compared to the uncoated olives by Day 21. This reduction in microbial growth underscores the preservative efficacy of the NG mixtures, likely resulting from the combined antimicrobial properties of CT and CV.

Importantly, the NGs did not inhibit the growth of lactic acid bacteria (LAB), which are crucial for the fermentation process. On the contrary, the LAB populations increased significantly in the presence of the NG mixtures, particularly NGCTCV 25:75. A study conducted by Papastolopoulou et al. [78] noted that, indeed, essential oil did not affect fermentation. This suggests that the polysaccharide 3D network in the NGs may provide a nutrient source for LAB, promoting their activity while, simultaneously, essential oil derivatives inhibit the growth of harmful bacteria. The dual effect of the novel examined NGs as natural preservative agents seems ideal for fermented products such as Chalkidiki PDO green table olives. To the best of our knowledge, this is the first time such a result has been reported, and further investigation is suggested. The implications of these results are significant in terms of fermented food preservation, as NGs can inhibit the survival of pathogens, while beneficial microorganisms are not affected by their presence.

Weight loss is a critical factor in maintaining the quality of Chalkidiki PDO green table olives during storage. The NG coatings, especially the NGCTCV 50:50 and NGCTCV 25:75 mixtures, were highly effective in controlling weight loss over the 21-day storage period. These coatings outperformed individual NGs, suggesting a synergistic effect that enhances moisture retention. This finding is consistent with other studies demonstrating the effectiveness of polysaccharide and essential oil-based coatings in reducing weight loss in fruits and vegetables [79,80,81,82,86]. By controlling the moisture levels, these coatings help preserve the physical and textural quality of the olives, making them attractive to consumers. A possible scenario of the mode of action could be the formation of a semi-permeable barrier. The hydrophilic properties of xanthan gum and kappa-carrageenan help retain moisture by bonding with water, while the nanoscale structure provides even coverage, reducing dehydration, which reduces the rate of evaporation [87].

The pH analysis further supported the effectiveness of the NGs, particularly in maintaining a stable, low pH throughout the storage period. The initial pH drop observed in the first week indicates active LAB fermentation, which is beneficial for the preservation process. The NGCTCV 50:50 and NGCTCV 75:25 mixtures were particularly effective in maintaining lower pH levels, likely due to the LAB activity and controlling yeast and mold populations. This stable pH environment is crucial for preventing the growth of pathogenic bacteria and ensuring the safety and quality of Chalkidiki PDO green table olives.

Color is a crucial quality parameter in the examined Chalkidiki PDO green table olives, and maintaining it during storage is critical for consumer acceptance. The L*a*b* colorimetry analysis demonstrated that the essential oil mixtures, particularly NGCTCV 25:75 and NGCTCV 50:50, were significantly more effective in preserving the color of the olives than individual NGs or uncoated samples. These mixtures effectively controlled the darkening of olives and retained the green and yellow hues, which are essential for the visual appeal of the product.

Finally, the mixed NG coatings, particularly NGCTCV 50:50 and NGCTCV 25:75, demonstrated superior preservation of sensory attributes such as taste, odor, color, and texture, outperforming both individual oil NGs and uncoated olives. This study is the first to report that combining essential oils in a NG formulation provides additional benefits for maintaining sensory qualities in fermented food products like Chalkidiki green table olives. All these findings indicate that the development of these novel NGs represents an ideal food technology for achieving the synergistic preservation of foods. Future research should focus on understanding the mechanisms of NGs’ mode of action and also testing more pathogens and conducting long-term stability studies. Investigating mechanisms, expanding sensory evaluations, and assessing costs and environmental impacts would be beneficial.

## 3. Conclusions

This study investigated the potential of innovative preservation techniques in advancing food technology, specifically using polysaccharide-based NGs enhanced with essential oils. These novel NGs constitute a natural potential alternative to synthetic preservative compounds, aligning with the demand for natural preservatives.

Specifically, the study explored the development, characterization, and application of novel polysaccharide-based NGs enhanced with essential oil derivatives CT and CV, examining a potential synergistic effect and, finally, their use as active edible coatings on fermented products such as Chalkidiki PDO green table olives for shelf life extension.

Physicochemical Stability: Zeta potential measurements confirmed the physicochemical stability of the NGs. Both DLS and SEM studies confirm that NGCT and NGCV samples showed an average particle size of ~34 (~70%) and ~310 nm (~100%), correspondingly, while the samples NGCTCV_50/50, NGCTCV_25/75, and NGCTCV_75/25 showed a major population of the particles between 140 nm and 480 nm and the rest in the range of μm.

Antioxidant and Antimicrobial Activity: Preliminary tests indicated the high antioxidant profile of all those coatings. Furthermore, a significant bactericidal effect was demonstrated by NG mixtures, while FIC confirmed the synergistic effect of NGCTCV 50:50 against *S. aureus* and NGCTCV 25:75 and NGCTCV 75:25 against *E. coli* O157:H7.

Shelf Life Extension: The application of NG coatings to Chalkidiki PDO green table olives demonstrated the efficacy of NGCTCV 50:50 and NGCTCV 25:75 in reducing weight loss, maintaining stable pH levels, and preserving the visual quality of the olives over the 21-day storage period. These NGs not only inhibited spoilage microorganisms but also did not affect the growth of beneficial lactic acid bacteria essential for fermentation.

Color and Sensory Preservation: Notwithstanding the fact that the L*a*b* colorimetry results showed that these NG formulations successfully prevented discoloration, maintaining the olives’ desired color characteristics, the mixtures seem to have statistically significant differences. Finally, the NGs managed to extend the olives’ shelf life over 14 days. The mixed NG coatings, particularly NGCTCV 50:50 and 25:75, effectively preserved the sensory qualities of Chalkidiki green table olives, outperforming both individual NGs and the uncoated samples. Overall, this research highlights the advantages of polysaccharide-based NGs, particularly in the effective ratios of 50:50 and 25:75, for extending the shelf life of perishable products like Chalkidiki PDO green table olives. These nanoemulgel ratios make them suitable for extending the shelf life of perishable products while maintaining their quality. The use of natural ingredients aligns with modern consumer trends towards clean label and sustainable preservation methods.

Future research should focus on the application of these NGs to a broader range of food products and under varying storage conditions. Additionally, further investigation into the mode of action of the synergistic effects of CT and CV could provide deeper insights into optimizing these formulations.

## 4. Materials and Methods

### 4.1. Materials

CT (CAS No. 5392-40-5), CV (CAS No. 499-75-2), Tween 80 (CAS No. 9005-65-6), xanthan gum (CAS No. 11138-66-2), and kappa-carrageenan (CAS No. 11114-20-8) were purchased from Sigma-Aldrich Co. (3050 Spruce Street, St. Louis, MO, USA, 63103; Tel.: 314-7715765). The purity of all the above-listed chemicals was higher than 99% (≥99% purity).

Müeller–Hinton agar plates, Müeller–Hinton broth, Tryptic Soy Agar (TSA), De Man–Rogosa–Sharpe agar (MRS), and sterile swabs and forceps were also acquired from Sigma-Aldrich Co. (3050 Spruce Street, St. Louis, MO, USA, 63103; 149 Tel.: 314-771-5765). Pure Gram-positive and Gram-negative bacterial cultures of *Staphylococcus aureus* and *E. coli O157:H7* were obtained from the Institute of Technology of Agricultural Products, ELGO151 DEMETER, Lykovryssi, Greece.

### 4.2. Preparation of NG Samples

**Figure 6:**
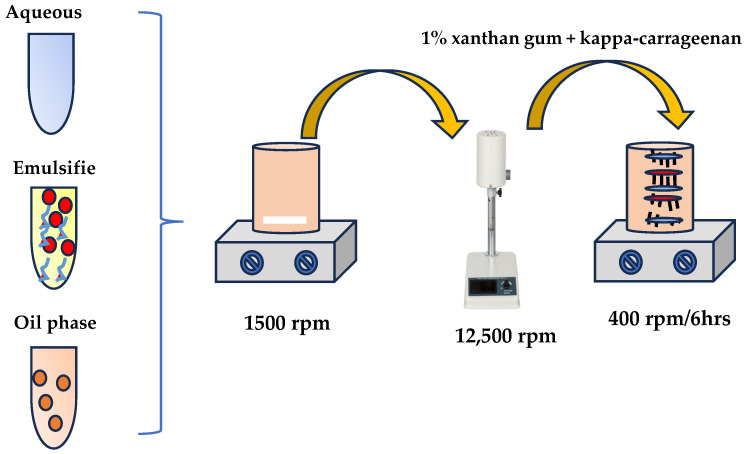
Schematic presentation of the NG preparation steps.

The preparation of all NGs was carried out according to the methodology suggested by Bahloul et al. [88], with some modifications. In detail, the preparation of the NGs commenced with the formulation of nanoemulsions by emulsifying the aqueous phase (H_2_O) with the oil phase, which consisted of EO derivatives and the emulsifier Tween 80. This process yielded a nanoemulsion with a final concentration of 2.5% solution. Five samples were prepared, including pure CT; CV; and their mixtures at ratios of 25:75, 50:50, and 75:25. Emulsification was performed using a high-speed ultrasonic homogenizer operating at 15,000 rpm for 20 min, with the emulsions maintained in an ice bath to avoid thermal instability. The emulsions were subsequently stored at 4 °C for 24 h to further enhance stability. Following this, xanthan gum and kappa-carrageenan were incorporated at the final stage to avoid clots at a concentration of 1%, and the mixture was homogenized by stirring at 400 rpm for over 6 h, leading to formulation of the NGs. In Figure 6, the preparation process is illustrated as followed step by step.

### 4.3. Physicochemical Characterization of the Obtained NEs

#### 4.3.1. Z-Potential Measurements

A NanoBrook Omni series (Brookhaven Instruments Corporation, 750 Blue Point Road, Holtsville, NY, USA, 11742) size and zeta potential analyzer equipped with a solvent-resistant electrode was used to determine the zeta potential of the NGs. The NGs were diluted 100 times with deionized water to reduce the multiple scattering effect and tested at 25 °C with an equilibrium of 120 s.

#### 4.3.2. Dynamic Light Scattering (DLS)

Dynamic Light Scattering (DLS) analyses were performed utilizing a sophisticated dual-angle size and molecular analysis system (Zetasizer Nano ZS, Malvern, UK) functioning at a wavelength of 633 nm with a helium-neon laser at the conventional angle of 173°. A square-section glass cuvette facilitated these analyses. These procedures aimed to determine the solutions’ hydrodynamic diameter and polydispersity index at a controlled temperature of 37 °C, employing the Einstein–Stokes equation for calculating the hydrodynamic radius (RH).

(1) $$R_{H}=\frac{k_{B^{T}}}{6\pi \eta D}$$

where kB (J/K) is the Boltzmann constant, T (K) is the absolute temperature, η (N·s·m^−2^) is the solvent’s dynamic viscosity, and D (m2·s^−1^) is the diffusion coefficient. The concentration of the solutions was approximately 0.1 wt%. To avoid aggregation phenomena, all obtained NGs dispersed in deionized water prior to measurement.

#### 4.3.3. Transmission Electron Microscope (SEM) Images of NEs

The surface morphologies of the samples were observed using a JEOL JSM-6510 LV SEM microscope (Ltd., Tokyo, Japan) equipped with an X-Act EDS detector from Oxford Instruments, Abingdon, Oxfordshire, UK (an acceleration voltage of 20 kV was applied), with a possibility to function under low-vacuum conditions. The SEM samples were prepared by mixing one dilute drop of prepared NGs dispersed in 5 mL acetone to give a slightly turbid solution on the copper grid and allowing it to dry well. Before examination, all samples were sputter-coated with gold/palladium for 45 s to prevent sample charging during observation with SEM.

### 4.4. Determination of the Antioxidant Activity of NGs with the 2,2-Diphenyl-1-Picrylhydrazyl (DPPH) Assay

The preparation of the DPPH ethanolic solution followed the methodology recommended by Kechagias et al. [89]. A specific quantity of 30 ppm DPPH radical was dissolved in 250 mL of ethanol to create a 2.16 mM DPPH radical solution. This solution was mixed using a vortex while avoiding light exposure, resulting in a deep purple solution with a stable pH of approximately 7.02 ± 0.01. To ensure its stability, the solution was stored at 4 °C for over 2 h before being used in the experiment. Serial dilutions were then performed to generate a calibration curve.

To examine the correlation between DPPH radical absorbance and concentration, a calibration graph was plotted. The initial ethanol-based DPPH radical solution was serially diluted with ethanol to prepare five different concentrations ranging from 0 to 60 mg/L (ppm). The absorbance of these diluted DPPH radical solutions was measured at a wavelength of 517 nm using a Jasco V-530 UV–Vis spectrometer [89]. A linear calibration curve was constructed from the absorbance data collected, following a previously described method, enabling quantitative analysis of the antioxidant capacity [26].

To assess the concentration required to achieve 50% antioxidant effectiveness (EC_50_) of the newly developed NG coatings NGCT, NGCV, and NGCTCV (25:75, 50:50, and 75:25), varying volumes of each coating (20, 40, 60, 80, and 100 μL) were incorporated, in triplicate, into a mixture containing 2.8 mL of a 30 ppm DPPH radical solution in ethanol and 0.2 mL of a sodium acetate buffer (CH_3_COONa·3H_2_O). A control sample consisting solely of the DPPH radical solution and buffer was also prepared for comparative analysis. After a one-hour reaction period, considered sufficient to reach equilibrium, the absorbance level was recorded at 517 nm for both the experimental and control coatings. Using a proper equation, the percentage of DPPH radical reduction at equilibrium for each coating was determined, allowing for the assessment of the antioxidant capacity of each sample.

(2) $$\%\;antioxidant\;activity\;\left(DPPH\right)=\frac{A_{0}^{517}-A_{sample}^{517}}{A_{0}^{517}}\times 100$$

The reduced percentage of remaining DPPH radical at equilibrium was inversely correlated with the increased antioxidant efficacy of the tested substance [26]. Based on this principle, the antioxidant activity percentages derived from the coatings NGCT, NGCV, and NGCTCV (25:75, 50:50, and 75:25) were graphically represented against the respective volumes of coatings used. The performance of these coatings was assessed using the equations derived from the graphs. By applying these linear equations, the EC_50,DPPH_ values, which indicate the concentration at which 50% antioxidant activity is observed, were accurately calculated for each sample. This process allowed accurate measurement of the antioxidant effectiveness and quantitative assessment of each NG.

### 4.5. Antibacterial Activity Test of the NGs

#### 4.5.1. Inhibition Zone Tests

The antimicrobial properties of the examined NGs, including NGCT, NGCV, and NGCTCV (25:75, 50:50, and 75:25), were assessed using the well diffusion method against two significant foodborne pathogens: *Escherichia coli* O157:H7 and *Staphylococcus aureus*. Initially, the bacterial strains were cultivated in Müeller–Hinton Broth at 37 °C for 24 h to reach a concentration of 10^7^ to 10^8^ CFU mL^−1^. The bacterial cultures were then spread onto Müller–Hinton agar plates using sterile cotton swabs, with the plates rotated every 60 degrees to ensure even bacterial distribution. A sterile cork borer, sanitized with alcohol and flame, was used to create 6-mm wells in the agar, which were subsequently filled with 100 μL of each treatment. Following this, the agar plates were incubated overnight at 37 °C. The effectiveness of the various treatments against the pathogens was assessed by measuring the sizes (in mm) of the clear zones surrounding each well using a digital caliper. These clear zones indicated regions where bacterial growth was inhibited by the NG coatings.

#### 4.5.2. MIC and MBC Tests and FIC Index

The Minimum Inhibitory Concentration (MIC) is identified as the least quantity of an antimicrobial agent needed to prevent visible microbial growth, indicating bacteriostatic capabilities without detailed analysis of the microbial viability. Cultures of *S. aureus* and *E. coli* O157:H7 were grown in Müeller–Hinton broth to achieve a bacterial concentration of 10^6^ CFU/mL. The antimicrobial efficacy of the examined NGs and their free EOs was assessed across a range of concentrations: 1000, 500, 250, 125, 62.5, 31.25, and 15.625 μg/mL. Later, serial dilutions of microbial cultures were prepared, and the antimicrobial agents were incorporated at each specified concentration for examination. The mixtures were then homogenized using a vortex mixer and incubated at 37 °C for a 24-h period. The control sample consisted of the microbial cultures without any coating, and it was also evaluated at a fixed concentration of 250 μg/mL. The presence of turbidity was monitored as an indication of microbial proliferation, with further confirmation obtained via culturing and colony enumeration. The procedure was performed in triplicate to ensure the reliability of the results.

To further assess the antimicrobial efficacy, the minimum bactericidal concentration (MBC) was determined by transferring 100 μL of the samples from the MIC test with no visible growth onto sterile Müeller–Hinton agar plates. These plates were incubated at 37 °C for 24 h. After incubation, the plates were assessed for bacterial growth. Finally, the MBC was determined as the lowest concentration at which no colonies were observed. This test was performed in triplicate to ensure consistent and reliable results.

To evaluate the potential synergistic effect between CT and CV in combination against the examined pathogens, the Fractional Inhibitory Concentration (FIC) index was calculated. The FIC index for each combination was calculated using the formula

(3) $$\mathrm{FIC}\;\mathrm{index}=\frac{MIC\;of\;CT\;\&\;CV}{MIC\;of\;CT}+\frac{MIC\;of\;CV\;\&\;CT}{MIC\;of\;CV}$$

If the FIC index is below 0.5, a synergistic effect is confirmed. If the FIC index ranges between 0.5 and 1.0, an additive effect is indicated. Finally, if the FIC index is above 1.0, an antagonistic effect occurs.

#### 4.5.3. Time-Killing Kinetics Assay

Time-killing assays were carried out according to the method described by Boonyanugomol et al. [90]. For these assays, the *E. coli* O157:H7 and *S. aureus* strains were standardized to a concentration of 10^6^ CFU/mL. The lowest MBC values of NGCTCV (25:75, 50:50, and 75:25) were used in the experiments. At intervals of 1 to 24 h, 50 μL samples were taken from the treated bacterial solutions. These samples were then subjected to ten-fold serial dilutions and plated on Mueller–Hinton Agar (MHA) for colony enumeration. A 0.5% *v*/*v* Tween 80 solution was employed as the control. The bacterial counts were plotted against time to create time-killing curves. All tests were performed in triplicate to ensure accuracy and reproducibility.

### 4.6. Application of NGs as Active Coatings in Chalkidiki Green Table Olives

All the manufactured NG treatments were applied as active coatings to preserve green table olives by the dipping method. Commercial Chalkidiki green table olives were purchased by a local market and stored at the laboratory, and then, 50 g of olives were weighted and dipped in 30 mL of each NG treatment, including NGCT, NGCV, and NGCTCV (25:75, 50:50, and 75:25) and left dipped for 30s to ensuring uniform coverage across all samples. Afterwards, every olive sample was wrapped carefully in a membrane and placed in a pot and stored in a dark place at room temperature. During the storage period, various analyses were performed at regular intervals to assess the effectiveness of the applied treatments in preserving Chalkidiki green table olives (see Figure S3). These analyses included pH measurements, L*a*b* colorimetry analysis, weight loss, and sensory evaluations.

#### 4.6.1. Physicochemical Properties of Chalkidiki Green Table Olives

The physicochemical properties of Chalkidiki green table olives coatings, including pH, color values (L*a*b*), and weight loss, were analyzed at specific intervals: Days 0, 7, 14, and 21.

The pH values of the Chalkidiki green table olive coatings were measured using a portable pH meter fitted with a penetration electrode and a temperature sensor (pH-Star, Matthäus GmbH, Poettmes, Germany). Prior to each set of measurements, the pH meter was calibrated using pH standard solutions of 4.0 and 7.0 and temperature-adjusted to match the olive coating temperature of 20 °C. The entire procedure was conducted in triplicate, and for each treatment group, ten separate pH readings were taken to ensure accuracy and reliability, as per the methods [91].

The weight loss analysis was conducted by measuring the initial weight of the olive samples NGCT, NGCV, and NGCTCV (25:75, 50:50, and 75:25) at the start of the experiment (Day 0) and at subsequent intervals on Days 7, 14, and 21. At each sampling point, the weight loss was determined by subtracting the final weight of the olives from their initial weight on the respective days. The percentage difference in weight was calculated and interpreted as humidity loss, serving as a quality indicator to assess significant weight loss among the examined samples.

##### L*a*b* Analysis

The alterations in the CIELAB color parameters (L*, a*, and b*) of Chalkidiki green table olives over a period of 9 days under refrigerated storage were assessed using a LS171 colorimeter from Linshang Company. Prior to conducting the measurements, the colorimeter was calibrated with a white standard plate in accordance with the methods cited in studies [92,93]. Color evaluations were conducted directly on the surface of the olive coatings, with each treatment group comprising three separate portions. For each of these portions, five discrete readings were taken to capture a robust assessment of the color. The total color differences (*ΔE*) were calculated using the equation

(4) $$\Delta E=\sqrt{{\left(L^{*}-L_{0}^{*}\right)}^{2}+{\left(a^{*}-a_{0}^{*}\right)}^{2}+{\left(b^{*}-b_{0}^{*}\right)}^{2}}$$

In this equation, L*_0_, a*_0_, and b*_0_ denote the initial color parameters of the Chalkidiki green table olives at Day 0 post-treatment. L*, a*, and b* represent the respective color parameters at different time points during the 21-day refrigerated storage at 4 °C.

#### 4.6.2. Evaluation of Bacterial Counts in Chalkidiki Green Table Olives

Chalkidiki green table olives were submitted to assessment for mesophilic bacteria, LAB bacteria, and yeasts and molds on Days 0, 7, 14, and 21 during the storage period at 4 °C. Initially a 10 g Chalkidiki green table olive sample was aseptically transferred to a stomacher bag, followed by homogenization with 90 mL of peptone water. Subsequently, a serial dilution (1:10 ratio) was performed.

For LAB bacteria, MRS agar was used, and 1 mL of the diluted sample was incorporated into the plates. The plates were then incubated anaerobically at 30 °C for 72 h following the guidelines of ISO 15214:1998 [94]. For the enumeration of yeasts and molds, Dichloran Rose Bengal Chloramphenicol (DRBC) agar was used. A 0.1 mL aliquot from the diluted samples was spread onto the DRBC agar plates, which were then incubated aerobically at 25 °C for 5 days, according to ISO 21527-1:2008 [95].

#### 4.6.3. Sensory Evaluation of Chalkidiki Green Table Olives

A sensory analysis was performed on Chalkidiki green table olives treated with NG coatings to evaluate key sensory characteristics, including taste, odor, color, texture, and overall acceptability. A panel of 15 trained assessors with expertise in food sensory evaluation participated in the assessment. An introductory session on the examined attributes to be assessed was performed, such as taste, odor, color, and texture, in order for the panelists to be familiarized with the sensory terminology used. The evaluation employed a 5-point hedonic scale, where a score of 5 indicated “like extremely” and a score of 1 indicated “dislike extremely”. An acceptability threshold score of 3.5 was used, as outlined by Hasani-Javanmardi et al. [96].

Each treatment group, including individual NGs (NGCV and NGCT); mixed NG coatings (NGCTCV 25:75, NGCTCV 50:50, and NGCTCV 75:25); and uncoated olives, was assessed at four different time points (Days 0, 7, 14, and 21). Each sample underwent triplicate assessments per replicate, resulting in a total of six evaluations for each treatment group at each time point. This allowed for a comprehensive analysis of the sensory attributes of the olives throughout the storage period. The results of this sensory evaluation provided insights into the effectiveness of the NG coatings in preserving the quality and sensory appeal of the olives over time.

### 4.7. Statistical Analysis

For the purposes of the statistical analysis, the average results of each experiment were recorded in triplicate. The software used refers to IBM SPSS version 22 (IBM Armonk, NY, USA), and the statistical analyses were performed. After checking for homogeneity of the data, an alpha level of *p* < 0.05 and, due to the low number of samples, a Kruskal–Wallis test was preferred as a tool for the examination of the significance of the resulting difference.

## Figures and Tables

**Table 1:** Zeta potential of the NG coatings.

Sample Code Name	Zeta Potential (mV)	Standard Error (SE)
NGCT	−42.23 ^A^	2.93
NGCV	−45.72 ^A^	2.79
NGCTCV25:75	−37.00 ^A^	1.76
NGCTCV50:50	−35.28 ^A^	3.70
NGCTCV75:25	−36.52 ^A^	2.03

^A^ Different letters indicate statistical significance at the 5% level (see Table S1).

**Table 2:** Mean EC_50_ values for NGs according to the DPPH and ABTS assay methods.

Sample Code Name	EC_50, DPPH_ (μL)	EC_50, ABTS_ (μL)
NGCT	224.28 ^A^	265.45 ^A^
NGCV	77.16 ^B^	101.40 ^B^
NGCTCV25:75	89.61 ^B^	118.60 ^B^
NGCTCV50:50	99.16 ^B^	128.20 ^B^
NGCTCV75:25	123.05 ^B^	155.30 ^B^

^A,B^ Different letters indicate statistical significance at the 5% level (see Table S2).

**Table 3:** Agar diffusion zone of the examined NGs against pathogens *S. aureus* and *E. coli* O157:H7.

Samples	*S. aureus* (in mm)	*E coli* O157:H7 (in mm)
NGCT	11.0 ± 0.1 ^A^	9.0 ± 0.1 ^a^
NGCV	15.0 ± 0.2 ^B^	12.0 ± 0.1 ^b^
NGCTCV25:75	16.5 ± 0.1 ^C^	12.3 ± 0.1 ^c^
NGCTCV50:50	20.1 ± 0.1 ^D^	15.0 ± 0.1 ^d^
NGCTCV75:25	18.3 ± 0.1 ^C^	12.05 ± 0.1 ^c^

The zones of inhibition were determined using the well diffusion method, and each value represents the average of three replicates (see Figure S1). ^A,B,C,D^ Means within a column for *Staphylococcus aureus*, followed by the same letter (A–D), are not significantly different at the *p* < 0.05 level, according to the pairwise comparison of treatments (see Table S3). ^a,b,c,d^ Means within a column for *Escherichia coli*, followed by the same letter (a–d), are not significantly different at the *p* < 0.05 level, according to the pairwise comparison of treatments (see Table S4).

**Table 4:** Antimicrobial effectiveness of different compounds against *E. coli* O157 and *S. aureus*.

Samples	MIC		MBC		FIC	
	*E. coli* O157:H7	*S. aureus*	*E. coli* O157:H7	*S. aureus*	*E. coli* O157:H7	*S. aureus*
NGCT	<1000 μg	<1000 μg	<1000 μg	<1000 μg		
NGCV	<500 μg	<500 μg	<500 μg	<500 μg		
NGCTCV25:75	<125 μg	<250 μg	<125 μg	<250 μg	0.375 (Synergy < 0.5)	0.75 (Additive < 1.00)
NGCTCV50:50	<250 μg	<125 μg	<250 μg	<125 μg	0.75 (Additive < 1.00)	0.375 (Synergy < 0.5)
NGCTCV75:25	<125 μg	<250 μg	<125 μg	<250 μg	0.375 (Synergy < 0.5)	0.75 (Additive < 1.00)

**Table 5:** Weight reduction of Chalkidiki green table olives coated with various NGs.

Weight Reduction	Uncoated	NGCT	NGCV	NGCTCV 25:75	NGCTCV 50:50	NGCTCV 75:25
Day 7	0.7% ^Ac^	0.2% ^Ab^	0.4% ^Abc^	0.1% ^Aa^	0.1% ^Aa^	0.2% ^Ab^
Day 14	4.6% ^Bc^	0.4% ^Aab^	1.3% ^Aab^	0.2% ^Aa^	0.3% ^Aa^	0.3% ^Aa^
Day 21	7.6% ^Bc^	1.2% ^Aab^	3.5% ^Ab^	0.3% ^Aa^	0.3% ^Aa^	0.5% ^Aa^

^AaBbc^ Values with different small letters within the same row indicate statistically significant differences at the 5% level (see Table S7). ^AaBbc^ Values with different caps letters within the same column indicate statistically significant differences at the 5% level (see Table S8).

**Table 6:** pH analysis of Chalkidiki green table olives over a 21-day storage period with NG coatings.

Days	Uncoated	NGCT	NGCV	NGCTCV 25:75	NGCTCV 50:50	NGCTCV 75:25
Day 0	4.3 (±0.01) ^Aa^	4.3 (±0.03) ^Aa^	4.3 (±0.02) ^Aa^	4.3 (±0.01) ^Aa^	4.3 (±0.02) ^Aa^	4.3 (±0.02) ^Aa^
Day 7	4.25 (±0.02) ^Aa^	4.15 (±0.03) ^Aa^	4.1 (±0.02) ^Bb^	3.79 (±0.02) ^Bc^	3.77 (±0.02) ^Cc^	3.81 (±0.03) ^Bb^
Day 14	4.65 (±0.02) ^Aa^	4.69 (±0.01) ^Bb^	4.67 (±0.02) ^Bb^	4.63 (±0.02) ^Cc^	4.59 (±0.03) ^Cc^	4.64 (±0.01) ^Cb^
Day 21	4.77 (±0.01) ^Aa^	4.67 (±0.02) ^Bb^	4.63 (±0.02) ^Bb^	4.69 (±0.02) ^Bb^	4.71 (±0.03) ^Cc^	4.77 (±0.02) ^Cc^

^AaBbCc^ Values with different letters within the same row indicate statistically significant differences at the 5% level (see Table S9). ^AaBbCc^ Values with different caps letters within the same column indicate statistically significant differences at the 5% level (see Table S10).

**Table 7:** Changes in L values of Chalkidiki green table olives coated with the examined NGs during 21 days of storage.

Days	NGCT	NGCV	NGCTCV 25:75	NGCTCV 50:50	NGCTCV 75:25	Uncoated
Day 0	51.97 ^Aa^	51.97 ^Aa^	51.97 ^Aa^	51.97 ^Aa^	51.97 ^Aa^	51.97 ^Aa^
Day 7	49.31 ^Bb^	47.40 ^Bc^	48.22 ^Aa^	49.98 ^Bd^	47.67 ^Aa^	47.75 ^Bb^
Day 14	46.87 ^Bb^	43.47 ^Bc^	48.88 ^Ad^	46.43 ^Ba^	47.99 ^Ab^	45.15 ^Ba^
Day 21	45.56 ^Bb^	41.45 ^Bc^	46.65 ^Bd^	45.68 ^Ba^	47.41 ^Ba^	41.52 ^Bb^

^AaBbcd^ Values within the same row (day) with different capital letters (A–D) are significantly different at the 5% level, indicating differences between treatments (see Table S11). ^AaBbcd^ Values within the same column (treatment) with different small letters (a–d) are significantly different at the 5% level, indicating changes over time for the same treatment (see Table S12).

**Table 8:** Changes in a* values of Chalkidiki green table olives coated with the examined NGs during 21 days of storage.

Days	NGCT	NGCV	NGCTCV 25:75	NGCTCV 50:50	NGCTCV 75:25	Uncoated
Day 0	4.21 ^Aa^	4.21 ^Aa^	4.21 ^Aa^	4.21 ^Aa^	4.21 ^Aa^	4.21 ^Aa^
Day 7	5.37 ^Aa^	6.74 ^Bb^	4.76 ^Aa^	4.65 ^Aa^	4.69 ^Aa^	5.52 ^Aa^
Day 14	5.17 ^Ba^	5.72 ^Bb^	6.48 ^Bb^	5.11 ^Bb^	5.53 ^Bb^	3.88 ^Bc^
Day 21	3.96 ^Ba^	6.71 ^Aa^	4.77 ^Aa^	4.36 ^Aa^	4.69 ^Aa^	4.04 ^Aa^

^AaBbc^ Values within the same row (day) with different capital letters (A–D) are significantly different at the 5% level, indicating differences between treatments (see Table S13). ^AaBbc^ Values within the same column (treatment) with different small letters (a–d) are significantly different at the 5% level, indicating changes over time for the same treatment (see Table S14).

**Table 9:** Changes in b values of Chalkidiki green table olives coated with the examined NGs during 21 days of storage.

Days	NGCT	NGCV	NGCTCV 25:75	NGCTCV 50:50	NGCTCV 75:25	Uncoated
Day 0	31.25 ^Aa^	31.25 ^Aa^	31.25 ^Aa^	31.25 ^Aa^	31.25 ^Aa^	31.25 ^Aa^
Day 7	26.05 ^Aa^	25.13 ^Aa^	25.12 ^Bb^	26.97 ^Aa^	28.28 ^Ab^	23.63 ^Bb^
Day 14	24.91 ^Aa^	19.73 ^Bb^	25.46 ^Ba^	23.89 ^Aa^	24.51 ^Aa^	20.49 ^Ba^
Day 21	24.04 ^Ab^	14.85 ^Bb^	23.62 ^Aa^	22.87 ^Aa^	23.73 ^Aa^	16.33 ^Ba^

^AaBb^ Values within the same row (day) with different capital letters (A–D) are significantly different at the 5% level, indicating differences between treatments (see Table S15). ^AaBb^ Values within the same column (treatment) with different small letters (a–d) are significantly different at the 5% level, indicating changes over time for the same treatment (see Table S16).

**Table 10:** Sensory analysis results for NG coatings on Chalkidiki green table olives over the examined period.

Day	NGCV	NGCT	NGCTCV 25:75	NGCTCV 50:50	NGCTCV 75:25	Uncoated
**Taste**						
Day 0	5.00 ± 0.2 ^Aa^	5.00 ± 0.2 ^Aa^	5.00 ± 0.2 ^Aa^	5.00 ± 0.2 ^Aa^	5.00 ± 0.2 ^Aa^	5.00 ± 0.2 ^Aa^
Day 7	4.23 ± 0.3 ^Bb^	4.61 ± 0.2 ^Bb^	4.84 ± 0.1 ^Ab^	4.93 ± 0.1 ^Ab^	4.87 ± 0.1 ^Ab^	3.99 ± 0.3 ^Bb^
Day 14	3.60 ± 0.2 ^Bc^	4.07 ± 0.2 ^Bc^	4.51 ± 0.1 ^Ac^	4.70 ± 0.1 ^Ac^	4.49 ± 0.2 ^Ac^	3.20 ± 0.3 ^Cc^
Day 21	2.87 ± 0.3 ^Cd^	3.53 ± 0.2 ^Bd^	4.11 ± 0.1 ^Ad^	4.24 ± 0.2 ^Ad^	4.07 ± 0.2 ^Ad^	1.00 ± 0.3 ^Dd^
**Odor**						
**Day**	**NGCV**	**NGCT**	**NGCTCV 25:75**	**NGCTCV 50:50**	**NGCTCV 75:25**	**Uncoated**
Day 0	5.00 ± 0.2 ^Aa^	5.00 ± 0.2 ^Aa^	5.00 ± 0.2 ^Aa^	5.00 ± 0.2 ^Aa^	5.00 ± 0.2 ^Aa^	5.00 ± 0.2 ^Aa^
Day 7	4.23 ± 0.2 ^Bb^	4.61 ± 0.2 ^Bb^	4.84 ± 0.1 ^Ab^	4.93 ± 0.1 ^Ab^	4.87 ± 0.1 ^Ab^	3.96 ± 0.3 ^Bb^
Day 14	3.60 ± 0.3 ^Bc^	4.49 ± 0.2 ^Ac^	4.74 ± 0.1 ^Ac^	4.73 ± 0.1 ^Ac^	4.71 ± 0.1 ^Ac^	2.67 ± 0.3 ^Cc^
Day 21	2.87 ± 0.2 ^Cd^	3.73 ± 0.2 ^Bd^	4.13 ± 0.1 ^Ad^	4.24 ± 0.1 ^Ad^	4.21 ± 0.2 ^Ad^	1.00 ± 0.2 ^Dd^
**Color**						
**Day**	**NGCV**	**NGCT**	**NGCTCV 25:75**	**NGCTCV 50:50**	**NGCTCV 75:25**	**Uncoated**
Day 0	5.00 ± 0.2 ^Aa^	5.00 ± 0.2 ^Aa^	5.00 ± 0.2 ^Aa^	5.00 ± 0.2 ^Aa^	5.00 ± 0.2 ^Aa^	5.00 ± 0.2 ^Aa^
Day 7	3.06 ± 0.2 ^Bc^	4.83 ± 0.2 ^Ab^	4.40 ± 0.2 ^Bb^	4.73 ± 0.1 ^Ab^	4.50 ± 0.2 ^Bb^	2.61 ± 0.3 ^Bc^
Day 14	2.75 ± 0.3 ^Cd^	4.35 ± 0.2 ^Ac^	3.96 ± 0.1 ^Bc^	4.26 ± 0.2 ^Ac^	4.05 ± 0.1 ^Ac^	2.35 ± 0.2 ^Cd^
Day 21	2.47 ± 0.2 ^Dd^	3.67 ± 0.3 ^Bd^	3.81 ± 0.2 ^Ad^	3.93 ± 0.1 ^Ad^	3.79 ± 0.1 ^Ad^	2.07 ± 0.2 ^Dd^
**Texture**						
**Day**	**NGCV**	**NGCT**	**NGCTCV 25:75**	**NGCTCV 50:50**	**NGCTCV 75:25**	**Uncoated**
Day 0	5.00 ± 0.2 ^Aa^	5.00 ± 0.2 ^Aa^	5.00 ± 0.2 ^Aa^	5.00 ± 0.2 ^Aa^	5.00 ± 0.2 ^Aa^	5.00 ± 0.2 ^Aa^
Day 7	3.06 ± 0.2 ^Bc^	4.83 ± 0.2 ^Ab^	4.40 ± 0.1 ^Bb^	4.73 ± 0.2 ^Ab^	4.50 ± 0.2 ^Bb^	2.61 ± 0.3 ^Bc^
Day 14	2.75 ± 0.3 ^Cd^	4.35 ± 0.2 ^Ac^	3.96 ± 0.2 ^Bc^	4.26 ± 0.1 ^Ac^	4.05 ± 0.1 ^Ac^	2.35 ± 0.2 ^Cd^
Day 21	2.47 ± 0.2 ^Dd^	3.67 ± 0.3 ^Bd^	3.81 ± 0.1 ^Ad^	3.93 ± 0.1 ^Ad^	3.79 ± 0.2 ^Ad^	1.49 ± 0.3 ^Dd^
**Overall**						
**Day**	**NGCV**	**NGCT**	**NGCTCV 25:75**	**NGCTCV 50:50**	**NGCTCV 75:25**	**Uncoated**
Day 0	5.00 ± 0.2 ^Aa^	5.00 ± 0.2 ^Aa^	5.00 ± 0.2 ^Aa^	5.00 ± 0.2 ^Aa^	5.00 ± 0.2 ^Aa^	5.00 ± 0.2 ^Aa^
Day 7	3.84 ± 0.3 ^Bb^	4.69 ± 0.2 ^Ab^	4.70 ± 0.1 ^Ab^	4.86 ± 0.1 ^Aa^	4.75 ± 0.1 ^Ab^	3.52 ± 0.3 ^Bc^
Day 14	3.32 ± 0.2 ^Cc^	4.30 ± 0.2 ^Bc^	4.41 ± 0.1 ^Bc^	4.56 ± 0.1 ^Ac^	4.42 ± 0.1 ^Bc^	2.74 ± 0.3 ^Cd^
Day 21	2.74 ± 0.3 ^Dd^	3.64 ± 0.2 ^Bd^	4.02 ± 0.2 ^Ad^	4.14 ± 0.1 ^Ad^	4.02 ± 0.1 ^Ad^	1.36 ± 0.3 ^Dd^

^AaBbCcDd^ Values within the same row (day) with different capital letters (A–D) are significantly different at the 5% level, indicating differences between treatments (see Table S17). ^AaBbCcDd^ Values within the same column (treatment) with different small letters (a–d) are significantly different at the 5% level, indicating changes over time for the same treatment (see Table S18).

## Data Availability

The datasets generated for this study are available on request from the corresponding authors.

## Supplementary Materials

The following supporting information can be downloaded at https://www.mdpi.com/article/10.3390/gels10110722/s1: Figure S1: Agar diffusion zone of nanogels against pathogens (A) *E. coli* O157:H7 and (B) *S. aureus*; Figure S2: Application process of olives coatings on nanogels; Table S1: Statistical analysis (Kruskal–Wallis) of the Zeta potential results title; Table S2: Statistical analysis (Kruskal–Wallis) for the antioxidant activity EC_50_ results; Table S3: Statistical analysis results (Kruskal–Wallis) for the *E. coli* O157:H7 antibacterial activity tests; Table S4: Statistical analysis results (Kruskal–Wallis) for *S. aureus* antibacterial activity tests; Table S5: Statistical analysis results (Kruskal–Wallis) for the yeast results; Table S6: Statistical analysis results (Kruskal–Wallis) for the LAB results; Table S7: Statistical analysis results (Kruskal–Wallis) for the weight loss results; Table S8: Statistical analysis results (Kruskal–Wallis) for the weight loss results; Table S9: Statistical analysis results (Kruskal–Wallis) for the pH results; Table S10: Statistical analysis results (Kruskal–Wallis) for the pH results; Table S11: Statistical analysis results (Kruskal–Wallis) for the L* parameter results; Table S12: Statistical analysis results (Kruskal–Wallis) for L* parameter results; Table S13. Statistical analysis results (Kruskal–Wallis) for the a* parameter results; Table S14: Statistical analysis results (Kruskal–Wallis) for the a* parameter results, Table S15: Statistical analysis results (Kruskal–Wallis) for the b* parameter results; Table S16: Statistical analysis results (Kruskal–Wallis) for b* parameter results; Table S17: Statistical analysis results (Kruskal-Wallis) for sensory analysis results; Table S18: Statistical analysis results (Kruskal–Wallis) for sensory analysis results.
