# Supplementary material for: Investigating the Synergistic Effects of Carvacrol and Citral-Edible Polysaccharide-Based Nanoemulgels on Shelf Life Extension of Chalkidiki Green Table Olives
Source: Gels. 2024 Nov 8;10(11):722. doi: 10.3390/gels10110722 (PMC11594034; doi:10.3390/gels10110722)
Supplement: Supplementary file 1 [file gels-10-00722-s001.zip › gels-3278291-supplementary.pdf]

Supplementary material for:

Investigating the Synergistic Effects of Carvacrol and Citral- edible Polysaccharide based Nanogels on shelf-life extension of Chalkidiki Green Table Olives.

Konstantinos Zaharioudakis[1], Constantinos E. Salmas [2,*], Nikolaos D. Andritsos[1], Areti A. Leontiou[1], Dimitrios Moschovas[2], Andreas Karydis-Messinis[2], Eleni Triantafyllou[2], Apostolos Avgeropoulos[2], Nikolaos E. Zafeiropoulos[2], Charalampos Proestos[3], and Aris E. Giannakas[1,*]

[1] Department of Food Science and Technology, University of Patras, 30100 Agrinio, Greece; zacharioudakis.k@upatras.gr (K.Z.); nandrits@upatras.gr (N.D.A.) aleontiu@upatras.gr (A.L.); gkechagi@upatras.gr (G.K.)

[2] Department of Material Science and Engineering, University of Ioannina, 45110 Ioannina, Greece; dmoschov@uoi.gr (D.M.); a.karyd@uoi.gr (A.K.-M.); triantafyllou.eleni@uoi.gr (E.T); aavger@uoi.gr (A.A.); nzafirop@uoi.gr (N.E.Z.)

[3] Laboratory of Food Chemistry, Department of Chemistry, National and Kapodistrian University of Athens Zografou, 15771 Athens, Greece; harpro@chem.uoa.gr (C.P.)

* Correspondence: ksalmas@uoi.gr (C.E.S.); agiannakas@upatras.gr (A.E.G.)

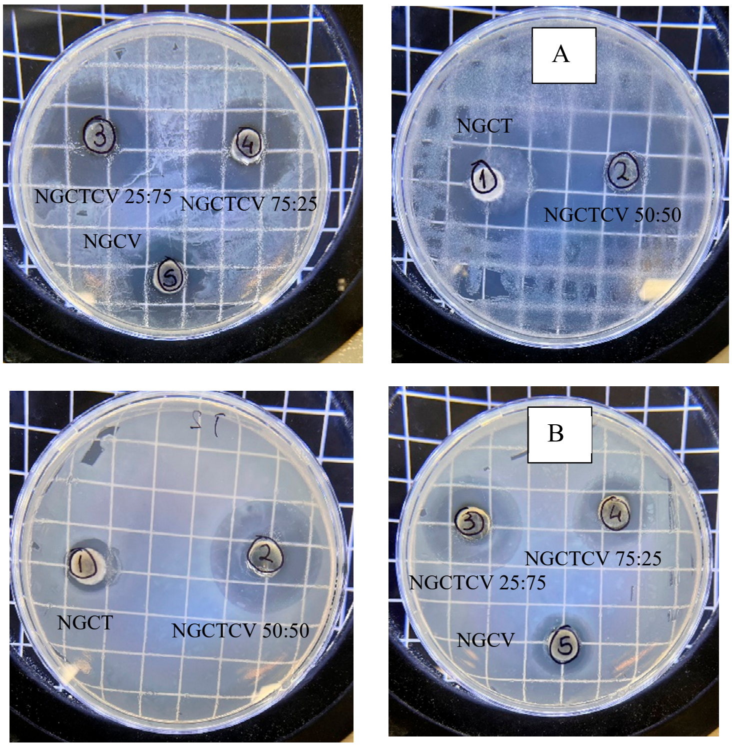

Figure S1. Agar Diffusion zone of nanogels against pathogens A) *E. coli O157:H7* and B) *S. aureus*

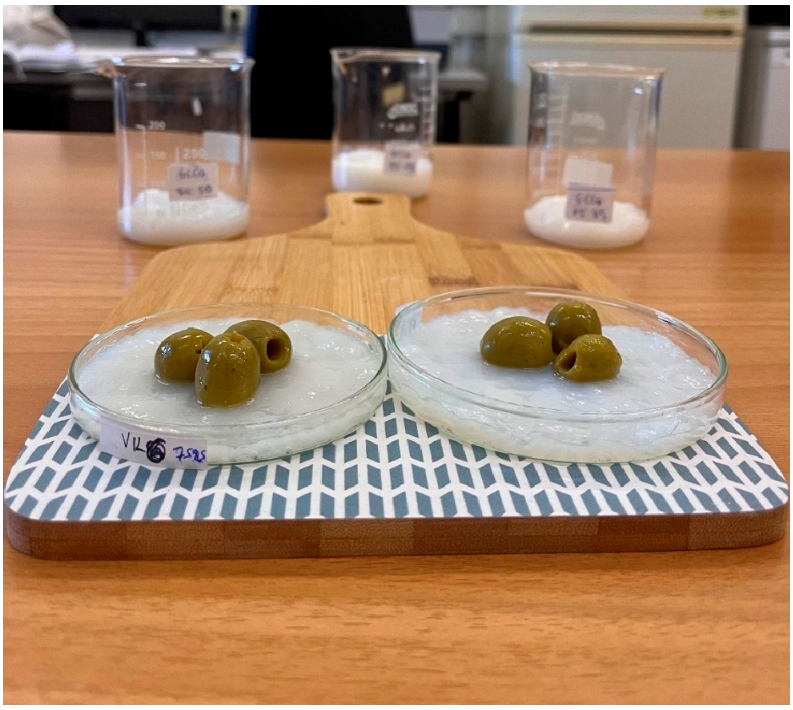

Figure S2. Application process of olives coatings on nanogels

Table S1. Statistical analysis (Kruskal-Wallis) of Zeta potential results

Pairwise Comparisons of Samples

Sample 1-Sample 2	Test Statistic	Std. Error	Std. Test Statistic	Sig.	Adj. Sig.[a]
Uncoated-NGCV	,333	4,359	,076	,939	1,000
Uncoated-NGCT	,667	4,359	,153	,878	1,000
Uncoated-NGCTCV75:25	2,000	4,359	,459	,646	1,000
Uncoated-NGCTCV50:50	4,667	4,359	1,071	,284	1,000
Uncoated-NGCTCV25:75	5,333	4,359	1,224	,221	1,000
NGCV-NGCT	,333	4,359	,076	,939	1,000
NGCV-NGCTCV75:25	-1,667	4,359	-,382	,702	1,000

NGCV-NGCTCV50:50	-4,333	4,359	-,994	,320	1,000
NGCV-NGCTCV25:75	-5,000	4,359	-1,147	,251	1,000
NGCT-NGCTCV75:25	-1,333	4,359	-,306	,760	1,000
NGCT-NGCTCV50:50	-4,000	4,359	-,918	,359	1,000
NGCT-NGCTCV25:75	-4,667	4,359	-1,071	,284	1,000
NGCTCV75:25-NGCTCV50:50	2,667	4,359	,612	,541	1,000
NGCTCV75:25-NGCTCV25:75	3,333	4,359	,765	,444	1,000
NGCTCV50:50-NGCTCV25:75	,667	4,359	,153	,878	1,000

Each row tests the null hypothesis that the Sample 1 and Sample 2 distributions are the same.

Asymptotic significances (2-sided tests) are displayed. The significance level is ,050.

a. Significance values have been adjusted by the Bonferroni correction for multiple tests.

Table S2. Statistical analysis (Kruskal-Wallis) for Antioxidant activity EC_{50} results

Pairwise Comparisons of Samples

Sample 1-Sample 2	Test Statistic	Std. Error	Std. Test Statistic	Sig.	Adj. Sig.[a]
NGCV-NGCTCV25:75	-3,000	3,651	-,822	,411	1,000
NGCV-NGCTCV50:50	-6,000	3,651	-1,643	,100	1,000
NGCV-NGCTCV75:25	-9,000	3,651	-2,465	,014	,137
NGCV-NGCT	12,000	3,651	3,286	,001	,010
NGCTCV25:75-NGCTCV50:50	-3,000	3,651	-,822	,411	1,000
NGCTCV25:75-NGCTCV75:25	-6,000	3,651	-1,643	,100	1,000
NGCTCV25:75-NGCT	9,000	3,651	2,465	,014	,137
NGCTCV50:50-NGCTCV75:25	-3,000	3,651	-,822	,411	1,000
NGCTCV50:50-NGCT	6,000	3,651	1,643	,100	1,000
NGCTCV75:25-NGCT	3,000	3,651	,822	,411	1,000

Each row tests the null hypothesis that the Sample 1 and Sample 2 distributions are the same.

Asymptotic significances (2-sided tests) are displayed. The significance level is ,050.

a. Significance values have been adjusted by the Bonferroni correction for multiple tests.

Table S3. Statistical analysis results (Kruskal-Wallis) for *E. coli* O157:H7 antibacterial activity tests

Pairwise Comparisons of Samples

Sample 1-Sample 2	Test Statistic	Std. Error	Std. Test Statistic	Sig.	Adj. Sig.[a]
NGCT-NGCV	-3,000	3,651	-,822	,411	1,000
NGCT-NGCTCV25:75	-6,000	3,651	-1,643	,100	1,000
NGCT-NGCTCV75:25	-9,000	3,651	-2,465	,014	,137
NGCT-NGCTCV50:50	-12,000	3,651	-3,286	,001	,010
NGCV-NGCTCV25:75	-3,000	3,651	-,822	,411	1,000
NGCV-NGCTCV75:25	-6,000	3,651	-1,643	,100	1,000
NGCV-NGCTCV50:50	-9,000	3,651	-2,465	,014	,137
NGCTCV25:75-NGCTCV75:25	-3,000	3,651	-,822	,411	1,000
NGCTCV25:75-NGCTCV50:50	-6,000	3,651	-1,643	,100	1,000
NGCTCV75:25-NGCTCV50:50	3,000	3,651	,822	,411	1,000

Each row tests the null hypothesis that the Sample 1 and Sample 2 distributions are the same.

Asymptotic significances (2-sided tests) are displayed. The significance level is ,050.

a. Significance values have been adjusted by the Bonferroni correction for multiple tests.

Table S4. Statistical analysis results (Kruskal-Wallis) for *S. aureus* antibacterial activity tests

Pairwise Comparisons of Samples

Sample 1-Sample 2	Test Statistic	Std. Error	Std. Test Statistic	Sig.	Adj. Sig.[a]
NGCT-NGCV	-4,833	3,648	-1,325	,185	1,000
NGCT-NGCTCV75:25	-4,833	3,648	-1,325	,185	1,000
NGCT-NGCTCV25:75	-8,333	3,648	-2,284	,022	,224
NGCT-NGCTCV50:50	-12,000	3,648	-3,289	,001	,010
NGCV-NGCTCV75:25	,000	3,648	,000	1,000	1,000
NGCV-NGCTCV25:75	-3,500	3,648	-,959	,337	1,000
NGCV-NGCTCV50:50	-7,167	3,648	-1,964	,049	,495
NGCTCV75:25-NGCTCV25:75	3,500	3,648	,959	,337	1,000
NGCTCV75:25-NGCTCV50:50	7,167	3,648	1,964	,049	,495
NGCTCV25:75-NGCTCV50:50	-3,667	3,648	-1,005	,315	1,000

Each row tests the null hypothesis that the Sample 1 and Sample 2 distributions are the same.

Asymptotic significances (2-sided tests) are displayed. The significance level is ,050.

a. Significance values have been adjusted by the Bonferroni correction for multiple tests.

Yeasts & Molds

Day 0

Table S4. Statistical analysis results (Kruskal-Wallis) for Yeasts results

Hypothesis Test Summary

	**Null Hypothesis**	**Test**	**Sig.**[a,b]	Decision
1	The distribution of Day_0_Y is the same across categories of Samples.	Independent-Samples Kruskal-Wallis Test	1,000	Retain the null hypothesis.
2	The distribution of Day_7_Y is the same across categories of Samples.	Independent-Samples Kruskal-Wallis Test	,007	Reject the null hypothesis.
3	The distribution of Day_14_Y is the same across categories of Samples.	Independent-Samples Kruskal-Wallis Test	,006	Reject the null hypothesis.
4	The distribution of Day_21_Y is the same across categories of Samples.	Independent-Samples Kruskal-Wallis Test	,006	Reject the null hypothesis.
5	The distribution of Day_0_LAB is the same across categories of Samples.	Independent-Samples Kruskal-Wallis Test	1,000	Retain the null hypothesis.
6	The distribution of Day_7_LAB is the same across categories of Samples.	Independent-Samples Kruskal-Wallis Test	,004	Reject the null hypothesis.
7	The distribution of Day_14_LAB is the same across categories of Samples.	Independent-Samples Kruskal-Wallis Test	,004	Reject the null hypothesis.
8	The distribution of Day_21_LAB is the same across categories of Samples.	Independent-Samples Kruskal-Wallis Test	,004	Reject the null hypothesis.

a. The significance level is ,050.
b. Asymptotic significance is displayed.

Day 7

Pairwise Comparisons of Samples

Sample 1-Sample 2	Test Statistic	Std. Error	Std. Test Statistic	Sig.	Adj. Sig.[a]

NGCTCV50:50-NGCV	4,167	4,334	,961	,336	1,000
NGCTCV50:50-NGCTCV75:25	-4,833	4,334	-1,115	,265	1,000
NGCTCV50:50-NGCTCV25:75	9,000	4,334	2,077	,038	,568
NGCTCV50:50-NGCT	13,500	4,334	3,115	,002	,028
NGCTCV50:50-Uncoated	-13,500	4,334	-3,115	,002	,028
NGCV-NGCTCV75:25	-,667	4,334	-,154	,878	1,000
NGCV-NGCTCV25:75	-4,833	4,334	-1,115	,265	1,000
NGCV-NGCT	9,333	4,334	2,153	,031	,469
NGCV-Uncoated	-9,333	4,334	-2,153	,031	,469
NGCTCV75:25-NGCTCV25:75	4,167	4,334	,961	,336	1,000
NGCTCV75:25-NGCT	8,667	4,334	2,000	,046	,683
NGCTCV75:25-Uncoated	-8,667	4,334	-2,000	,046	,683
NGCTCV25:75-NGCT	4,500	4,334	1,038	,299	1,000
NGCTCV25:75-Uncoated	-4,500	4,334	-1,038	,299	1,000
NGCT-Uncoated	,000	4,334	,000	1,000	1,000

Each row tests the null hypothesis that the Sample 1 and Sample 2 distributions are the same.

Asymptotic significances (2-sided tests) are displayed. The significance level is ,050.

a. Significance values have been adjusted by the Bonferroni correction for multiple tests.

Day 14

Pairwise Comparisons of Samples

Sample 1-Sample 2	Test Statistic	Std. Error	Std. Test Statistic	Sig.	Adj. Sig.[a]
NGCTCV50:50-NGCTCV25:75	3,000	4,336	,692	,489	1,000
NGCTCV50:50-NGCV	6,000	4,336	1,384	,166	1,000
NGCTCV50:50-NGCTCV75:25	-9,000	4,336	-2,075	,038	,569
NGCTCV50:50-NGCT	13,500	4,336	3,113	,002	,028
NGCTCV50:50-Uncoated	-13,500	4,336	-3,113	,002	,028

NGCTCV25:75-NGCV	3,000	4,336	,692	,489	1,000
NGCTCV25:75-NGCTCV75:25	-6,000	4,336	-1,384	,166	1,000
NGCTCV25:75-NGCT	10,500	4,336	2,421	,015	,232
NGCTCV25:75-Uncoated	-10,500	4,336	-2,421	,015	,232
NGCV-NGCTCV75:25	-3,000	4,336	-,692	,489	1,000
NGCV-NGCT	7,500	4,336	1,730	,084	1,000
NGCV-Uncoated	-7,500	4,336	-1,730	,084	1,000
NGCTCV75:25-NGCT	4,500	4,336	1,038	,299	1,000
NGCTCV75:25-Uncoated	-4,500	4,336	-1,038	,299	1,000
NGCT-Uncoated	,000	4,336	,000	1,000	1,000

Each row tests the null hypothesis that the Sample 1 and Sample 2 distributions are the same.

Asymptotic significances (2-sided tests) are displayed. The significance level is ,050.

a. Significance values have been adjusted by the Bonferroni correction for multiple tests.

Day 21

Pairwise Comparisons of Samples

Sample 1-Sample 2	Test Statistic	Std. Error	Std. Test Statistic	Sig.	Adj. Sig.[a]
NGCV-NGCTCV75:25	-3,000	4,336	-,692	,489	1,000
NGCV-NGCTCV50:50	-6,000	4,336	-1,384	,166	1,000
NGCV-NGCTCV25:75	-9,000	4,336	-2,075	,038	,569
NGCV-NGCT	13,500	4,336	3,113	,002	,028
NGCV-Uncoated	-13,500	4,336	-3,113	,002	,028
NGCTCV75:25-NGCTCV50:50	3,000	4,336	,692	,489	1,000
NGCTCV75:25-NGCTCV25:75	6,000	4,336	1,384	,166	1,000
NGCTCV75:25-NGCT	10,500	4,336	2,421	,015	,232

NGCTCV75:25-Uncoated	-10,500	4,336	-2,421	,015	,232
NGCTCV50:50-NGCTCV25:75	3,000	4,336	,692	,489	1,000
NGCTCV50:50-NGCT	7,500	4,336	1,730	,084	1,000
NGCTCV50:50-Uncoated	-7,500	4,336	-1,730	,084	1,000
NGCTCV25:75-NGCT	4,500	4,336	1,038	,299	1,000
NGCTCV25:75-Uncoated	-4,500	4,336	-1,038	,299	1,000
NGCT-Uncoated	,000	4,336	,000	1,000	1,000

Each row tests the null hypothesis that the Sample 1 and Sample 2 distributions are the same.

Asymptotic significances (2-sided tests) are displayed. The significance level is ,050.

a. Significance values have been adjusted by the Bonferroni correction for multiple tests.

Table S6. Statistical analysis results (Kruskal-Wallis) for LAB results

Day 7

Pairwise Comparisons of Samples

Sample 1-Sample 2	Test Statistic	Std. Error	Std. Test Statistic	Sig.	Adj. Sig.[a]
Uncoated-NGCV	3,000	4,180	,718	,473	1,000
Uncoated-NGCT	7,500	4,180	1,794	,073	1,000
Uncoated-NGCTCV50:50	7,500	4,180	1,794	,073	1,000
Uncoated-NGCTCV25:75	13,500	4,180	3,230	,001	,019
Uncoated-NGCTCV75:25	13,500	4,180	3,230	,001	,019
NGCV-NGCT	4,500	4,180	1,077	,282	1,000
NGCV-NGCTCV50:50	-4,500	4,180	-1,077	,282	1,000
NGCV-NGCTCV25:75	-10,500	4,180	-2,512	,012	,180

NGCV-NGCTCV75:25	-10,500	4,180	-2,512	,012	,180
NGCT-NGCTCV50:50	,000	4,180	,000	1,000	1,000
NGCT-NGCTCV25:75	-6,000	4,180	-1,435	,151	1,000
NGCT-NGCTCV75:25	-6,000	4,180	-1,435	,151	1,000
NGCTCV50:50-NGCTCV25:75	6,000	4,180	1,435	,151	1,000
NGCTCV50:50-NGCTCV75:25	-6,000	4,180	-1,435	,151	1,000
NGCTCV25:75-NGCTCV75:25	,000	4,180	,000	1,000	1,000

Each row tests the null hypothesis that the Sample 1 and Sample 2 distributions are the same.

Asymptotic significances (2-sided tests) are displayed. The significance level is ,050.

a. Significance values have been adjusted by the Bonferroni correction for multiple tests.

Day 14

LAB

Pairwise Comparisons of Samples

Sample 1-Sample 2	Test Statistic	Std. Error	Std. Test Statistic	Sig.	Adj. Sig.[a]
NGCT-Uncoated	,000	4,180	,000	1,000	1,000
NGCT-NGCV	-6,000	4,180	-1,435	,151	1,000
NGCT-NGCTCV25:75	-6,000	4,180	-1,435	,151	1,000
NGCT-NGCTCV50:50	-10,500	4,180	-2,512	,012	,180
NGCT-NGCTCV75:25	-13,500	4,180	-3,230	,001	,019
Uncoated-NGCV	6,000	4,180	1,435	,151	1,000
Uncoated-NGCTCV25:75	6,000	4,180	1,435	,151	1,000
Uncoated-NGCTCV50:50	10,500	4,180	2,512	,012	,180
Uncoated-NGCTCV75:25	13,500	4,180	3,230	,001	,019
NGCV-NGCTCV25:75	,000	4,180	,000	1,000	1,000
NGCV-NGCTCV50:50	-4,500	4,180	-1,077	,282	1,000

NGCV-NGCTCV75:25	-7,500	4,180	-1,794	,073	1,000
NGCTCV25:75-NGCTCV50:50	-4,500	4,180	-1,077	,282	1,000
NGCTCV25:75-NGCTCV75:25	-7,500	4,180	-1,794	,073	1,000
NGCTCV50:50-NGCTCV75:25	-3,000	4,180	-,718	,473	1,000

Each row tests the null hypothesis that the Sample 1 and Sample 2 distributions are the same.

Asymptotic significances (2-sided tests) are displayed. The significance level is ,050.

a. Significance values have been adjusted by the Bonferroni correction for multiple tests.

Day 21

Pairwise Comparisons of Samples

Sample 1-Sample 2	Test Statistic	Std. Error	Std. Test Statistic	Sig.	Adj. Sig.[a]
NGCT-NGCV	-4,500	4,243	-1,061	,289	1,000
NGCT-NGCTCV25:75	-4,500	4,243	-1,061	,289	1,000
NGCT-Uncoated	-9,000	4,243	-2,121	,034	,508
NGCT-NGCTCV75:25	-12,000	4,243	-2,828	,005	,070
NGCT-NGCTCV50:50	-15,000	4,243	-3,536	<,001	,006
NGCV-NGCTCV25:75	,000	4,243	,000	1,000	1,000
NGCV-Uncoated	-4,500	4,243	-1,061	,289	1,000
NGCV-NGCTCV75:25	-7,500	4,243	-1,768	,077	1,000
NGCV-NGCTCV50:50	-10,500	4,243	-2,475	,013	,200
NGCTCV25:75-Uncoated	-4,500	4,243	-1,061	,289	1,000
NGCTCV25:75-NGCTCV75:25	-7,500	4,243	-1,768	,077	1,000
NGCTCV25:75-NGCTCV50:50	-10,500	4,243	-2,475	,013	,200
Uncoated-NGCTCV75:25	3,000	4,243	,707	,480	1,000
Uncoated-NGCTCV50:50	6,000	4,243	1,414	,157	1,000

NGCTCV75:25-NGCTCV50:50	3,000	4,243	,707	,480	1,000

Each row tests the null hypothesis that the Sample 1 and Sample 2 distributions are the same.

Asymptotic significances (2-sided tests) are displayed. The significance level is ,050.

a. Significance values have been adjusted by the Bonferroni correction for multiple tests.

Table S7. Statistical analysis results (Kruskal-Wallis) for weight loss results

Hypothesis Test Summary

	Null Hypothesis	Test	Sig.[a,b]	Decision
1	The distribution of Weight_Day_7 is the same across categories of Samples_weight.	Independent-Samples Kruskal-Wallis Test	,014	Reject the null hypothesis.
2	The distribution of Weight_Day_14 is the same across categories of Samples_weight.	Independent-Samples Kruskal-Wallis Test	,031	Reject the null hypothesis.
3	The distribution of Weight_Day_21 is the same across categories of Samples_weight.	Independent-Samples Kruskal-Wallis Test	,024	Reject the null hypothesis.

a. The significance level is ,050.

b. Asymptotic significance is displayed.

Pairwise Comparisons of Samples_weight

Sample 1-Sample 2	Test Statistic	Std. Error	Std. Test Statistic	Sig.	Adj. Sig.[a]
NGCTCV 25:75-NGCTCV 50:50	-3,000	4,229	-,709	,478	1,000
NGCTCV 25:75-NGCT	4,833	4,229	1,143	,253	1,000
NGCTCV 25:75-NGCTCV 75:25	-4,833	4,229	-1,143	,253	1,000

NGCTCV 25:75-NGCV	10,667	4,229	2,522	,012	,175
NGCTCV 25:75-Uncoated	-13,667	4,229	-3,232	,001	,018
NGCTCV 50:50-NGCT	1,833	4,229	,434	,665	1,000
NGCTCV 50:50-NGCTCV 75:25	-1,833	4,229	-,434	,665	1,000
NGCTCV 50:50-NGCV	7,667	4,229	1,813	,070	1,000
NGCTCV 50:50-Uncoated	-10,667	4,229	-2,522	,012	,175
NGCT-NGCTCV 75:25	,000	4,229	,000	1,000	1,000
NGCT-NGCV	-5,833	4,229	-1,379	,168	1,000
NGCT-Uncoated	-8,833	4,229	-2,089	,037	,551
NGCTCV 75:25-NGCV	5,833	4,229	1,379	,168	1,000
NGCTCV 75:25-Uncoated	-8,833	4,229	-2,089	,037	,551
NGCV-Uncoated	-3,000	4,229	-,709	,478	1,000

Each row tests the null hypothesis that the Sample 1 and Sample 2 distributions are the same.

Asymptotic significances (2-sided tests) are displayed. The significance level is ,050.

a. Significance values have been adjusted by the Bonferroni correction for multiple tests.

Day 14

Pairwise Comparisons of Samples_weight

Sample 1-Sample 2	Test Statistic	Std. Error	Std. Test Statistic	Sig.	Adj. Sig.[a]
NGCTCV 50:50-NGCTCV 25:75	,667	4,291	,155	,877	1,000
NGCTCV 50:50-NGCTCV 75:25	-,667	4,291	-,155	,877	1,000
NGCTCV 50:50-NGCT	4,000	4,291	,932	,351	1,000
NGCTCV 50:50-NGCV	8,000	4,291	1,864	,062	,934
NGCTCV 50:50-Uncoated	-11,667	4,291	-2,719	,007	,098
NGCTCV 25:75-NGCT	3,333	4,291	,777	,437	1,000
NGCTCV 75:25-NGCT	3,333	4,291	,777	,437	1,000
NGCTCV 25:75-NGCV	7,333	4,291	1,709	,087	1,000
NGCTCV 75:25-NGCV	7,333	4,291	1,709	,087	1,000
NGCTCV 25:75-NGCTCV 75:25	,000	4,291	,000	1,000	1,000
NGCTCV 25:75-Uncoated	-11,000	4,291	-2,564	,010	,155
NGCTCV 75:25-Uncoated	-11,000	4,291	-2,564	,010	,155
NGCT-NGCV	-4,000	4,291	-,932	,351	1,000
NGCT-Uncoated	-7,667	4,291	-1,787	,074	1,000
NGCV-Uncoated	-3,667	4,291	-,855	,393	1,000

Each row tests the null hypothesis that the Sample 1 and Sample 2 distributions are the same.

Asymptotic significances (2-sided tests) are displayed. The significance level is ,050.

a. Significance values have been adjusted by the Bonferroni correction for multiple tests.

Day 21

Pairwise Comparisons of Samples_weight

Sample 1-Sample 2	Test Statistic	Std. Error	Std. Test Statistic	Sig.	Adj. Sig.[a]
NGCTCV 25:75-NGCTCV 50:50	-,333	4,327	-,077	,939	1,000
NGCTCV 25:75-NGCTCV 75:25	-,667	4,327	-,154	,878	1,000
NGCTCV 25:75-NGCT	3,667	4,327	,847	,397	1,000
NGCTCV 25:75-NGCV	8,667	4,327	2,003	,045	,678
NGCTCV 25:75-Uncoated	-11,667	4,327	-2,696	,007	,105
NGCTCV 50:50-NGCTCV 75:25	-,333	4,327	-,077	,939	1,000
NGCTCV 50:50-NGCT	3,333	4,327	,770	,441	1,000
NGCTCV 50:50-NGCV	8,333	4,327	1,926	,054	,812
NGCTCV 50:50-Uncoated	-11,333	4,327	-2,619	,009	,132
NGCTCV 75:25-NGCT	3,000	4,327	,693	,488	1,000

NGCTCV 75:25-NGCV	8,000	4,327	1,849	,064	,967
NGCTCV 75:25-Uncoated	-11,000	4,327	-2,542	,011	,165
NGCT-NGCV	-5,000	4,327	-1,155	,248	1,000
NGCT-Uncoated	-8,000	4,327	-1,849	,064	,967
NGCV-Uncoated	-3,000	4,327	-,693	,488	1,000

Each row tests the null hypothesis that the Sample 1 and Sample 2 distributions are the same.

Asymptotic significances (2-sided tests) are displayed. The significance level is ,050.

a. Significance values have been adjusted by the Bonferroni correction for multiple tests.

Table S8. Statistical analysis results (Kruskal-Wallis) for weight loss results

Hypothesis Test Summary

	Null Hypothesis	Test	Sig.[a,b]	Decision
1	The distribution of NGCV is the same across categories of Days_W.	Independent-Samples Kruskal-Wallis Test	,273	Retain the null hypothesis.
2	The distribution of NGCT is the same across categories of Days_W.	Independent-Samples Kruskal-Wallis Test	,025	Reject the null hypothesis.
3	The distribution of W_NGCTCV_25_75 is the same across categories of Days_W.	Independent-Samples Kruskal-Wallis Test	,047	Reject the null hypothesis.

4	The distribution of W_NGCTCV_50_50 is the same across categories of Days_W.	Independent-Samples Kruskal-Wallis Test	,047	Reject the null hypothesis.
5	The distribution of W_NGCTCV_75_25 is the same across categories of Days_W.	Independent-Samples Kruskal-Wallis Test	,025	Reject the null hypothesis.
6	The distribution of W_Uncoated is the same across categories of Days_W.	Independent-Samples Kruskal-Wallis Test	,025	Reject the null hypothesis.

a. The significance level is ,050.

b. Asymptotic significance is displayed.

Table S9. Statistical analysis results (Kruskal-Wallis) for pH results

Hypothesis Test Summary

	Null Hypothesis	Test	Sig.[a,b]	Decision
1	The distribution of pH_day0 is the same across categories of Samples_weight.	Independent-Samples Kruskal-Wallis Test	1,000	Retain the null hypothesis.
2	The distribution of pH_day7 is the same across categories of Samples_weight.	Independent-Samples Kruskal-Wallis Test	,004	Reject the null hypothesis.
3	The distribution of pH_day14 is the same across categories of Samples_weight.	Independent-Samples Kruskal-Wallis Test	,004	Reject the null hypothesis.

4	The distribution of pH_day21 is the same across categories of Samples_weight.	Independent-Samples Kruskal-Wallis Test	,004	Reject the null hypothesis.

a. The significance level is ,050.

b. Asymptotic significance is displayed.

Day 7

Pairwise Comparisons of Samples_weight

Sample 1-Sample 2	Test Statistic	Std. Error	Std. Test Statistic	Sig.	Adj. Sig.[a]
NGCTCV 50:50-NGCTCV 25:75	3,000	4,305	,697	,486	1,000
NGCTCV 50:50-NGCTCV 75:25	-6,000	4,305	-1,394	,163	1,000
NGCTCV 50:50-NGCV	9,000	4,305	2,091	,037	,548
NGCTCV 50:50-NGCT	12,000	4,305	2,788	,005	,080
NGCTCV 50:50-Uncoated	-15,000	4,305	-3,485	<,001	,007
NGCTCV 25:75-NGCTCV 75:25	-3,000	4,305	-,697	,486	1,000
NGCTCV 25:75-NGCV	6,000	4,305	1,394	,163	1,000
NGCTCV 25:75-NGCT	9,000	4,305	2,091	,037	,548
NGCTCV 25:75-Uncoated	-12,000	4,305	-2,788	,005	,080

NGCTCV 75:25-NGCV	3,000	4,305	,697	,486	1,000
NGCTCV 75:25-NGCT	6,000	4,305	1,394	,163	1,000
NGCTCV 75:25-Uncoated	-9,000	4,305	-2,091	,037	,548
NGCV-NGCT	3,000	4,305	,697	,486	1,000
NGCV-Uncoated	-6,000	4,305	-1,394	,163	1,000
NGCT-Uncoated	-3,000	4,305	-,697	,486	1,000

Each row tests the null hypothesis that the Sample 1 and Sample 2 distributions are the same.

Asymptotic significances (2-sided tests) are displayed. The significance level is ,050.

a. Significance values have been adjusted by the Bonferroni correction for multiple tests.

Day 14

Pairwise Comparisons of Samples_weight

Sample 1-Sample 2	Test Statistic	Std. Error	Std. Test Statistic	Sig.	Adj. Sig.[a]
NGCTCV 50:50-NGCTCV 25:75	3,000	4,305	,697	,486	1,000
NGCTCV 50:50-NGCTCV 75:25	-6,000	4,305	-1,394	,163	1,000
NGCTCV 50:50-Uncoated	-9,000	4,305	-2,091	,037	,548

NGCTCV 50:50-NGCV	12,000	4,305	2,788	,005	,080
NGCTCV 50:50-NGCT	15,000	4,305	3,485	<,001	,007
NGCTCV 25:75-NGCTCV 75:25	-3,000	4,305	-,697	,486	1,000
NGCTCV 25:75-Uncoated	-6,000	4,305	-1,394	,163	1,000
NGCTCV 25:75-NGCV	9,000	4,305	2,091	,037	,548
NGCTCV 25:75-NGCT	12,000	4,305	2,788	,005	,080
NGCTCV 75:25-Uncoated	-3,000	4,305	-,697	,486	1,000
NGCTCV 75:25-NGCV	6,000	4,305	1,394	,163	1,000
NGCTCV 75:25-NGCT	9,000	4,305	2,091	,037	,548
Uncoated-NGCV	3,000	4,305	,697	,486	1,000
Uncoated-NGCT	6,000	4,305	1,394	,163	1,000
NGCV-NGCT	3,000	4,305	,697	,486	1,000

Each row tests the null hypothesis that the Sample 1 and Sample 2 distributions are the same.

Asymptotic significances (2-sided tests) are displayed. The significance level is ,050.

a. Significance values have been adjusted by the Bonferroni correction for multiple tests.

Day 21

Pairwise Comparisons of Samples_weight

Sample 1-Sample 2	Test Statistic	Std. Error	Std. Test Statistic	Sig.	Adj. Sig.[a]
NGCV-NGCT	3,000	4,243	,707	,480	1,000
NGCV-NGCTCV 25:75	-6,000	4,243	-1,414	,157	1,000
NGCV-NGCTCV 50:50	-9,000	4,243	-2,121	,034	,508
NGCV-NGCTCV 75:25	-13,500	4,243	-3,182	,001	,022
NGCV-Uncoated	-13,500	4,243	-3,182	,001	,022
NGCT-NGCTCV 25:75	-3,000	4,243	-,707	,480	1,000
NGCT-NGCTCV 50:50	-6,000	4,243	-1,414	,157	1,000
NGCT-NGCTCV 75:25	-10,500	4,243	-2,475	,013	,200
NGCT-Uncoated	-10,500	4,243	-2,475	,013	,200
NGCTCV 25:75-NGCTCV 50:50	-3,000	4,243	-,707	,480	1,000
NGCTCV 25:75-NGCTCV 75:25	-7,500	4,243	-1,768	,077	1,000
NGCTCV 25:75-Uncoated	-7,500	4,243	-1,768	,077	1,000
NGCTCV 50:50-NGCTCV 75:25	-4,500	4,243	-1,061	,289	1,000
NGCTCV 50:50-Uncoated	-4,500	4,243	-1,061	,289	1,000
NGCTCV 75:25-Uncoated	,000	4,243	,000	1,000	1,000

Each row tests the null hypothesis that the Sample 1 and Sample 2 distributions are the same.

Asymptotic significances (2-sided tests) are displayed. The significance level is ,050.

a. Significance values have been adjusted by the Bonferroni correction for multiple tests.

Table S10. Statistical analysis results (Kruskal-Wallis) for pH results

Hypothesis Test Summary

	Null Hypothesis	Test	Sig.[a,b]	Decision
1	The distribution of pH_NGCV is the same across categories of Days.	Independent-Samples Kruskal-Wallis Test	,012	Reject the null hypothesis.
2	The distribution of pH_NGCT is the same across categories of Days.	Independent-Samples Kruskal-Wallis Test	,012	Reject the null hypothesis.
3	The distribution of pH_NGCTCV_25_75 is the same across categories of Days.	Independent-Samples Kruskal-Wallis Test	,012	Reject the null hypothesis.
4	The distribution of pH_NGCTCV_50_50 is the same across categories of Days.	Independent-Samples Kruskal-Wallis Test	,012	Reject the null hypothesis.
5	The distribution of pH_NGCTCV_75_25 is the same across categories of Days.	Independent-Samples Kruskal-Wallis Test	,012	Reject the null hypothesis.
6	The distribution of pH_Uncoated is the same across categories of Days.	Independent-Samples Kruskal-Wallis Test	,012	Reject the null hypothesis.

a. The significance level is ,050.

b. Asymptotic significance is displayed.

Table S11. Statistical analysis results (Kruskal-Wallis) for L* parameter results

	**Hypothesis Test Summary**			
	**Null Hypothesis**	**Test**	**Sig.**[a,b]	Decision
1	The distribution of L_day0 is the same across categories of color_samples.	Independent-Samples Kruskal-Wallis Test	1,000	Retain the null hypothesis.
2	The distribution of L_day7 is the same across categories of color_samples.	Independent-Samples Kruskal-Wallis Test	,029	Reject the null hypothesis.
3	The distribution of L_day14 is the same across categories of color_samples.	Independent-Samples Kruskal-Wallis Test	,007	Reject the null hypothesis.
4	The distribution of L_day21 is the same across categories of color_samples.	Independent-Samples Kruskal-Wallis Test	,008	Reject the null hypothesis.
5	The distribution of a_day0 is the same across categories of color_samples.	Independent-Samples Kruskal-Wallis Test	1,000	Retain the null hypothesis.
6	The distribution of a_day7 is the same across categories of color_samples.	Independent-Samples Kruskal-Wallis Test	,046	Reject the null hypothesis.
7	The distribution of a_day14 is the same across categories of color_samples.	Independent-Samples Kruskal-Wallis Test	,016	Reject the null hypothesis.
8	The distribution of a_day21 is the same across categories of color_samples.	Independent-Samples Kruskal-Wallis Test	,028	Reject the null hypothesis.
9	The distribution of b_day0 is the same across categories of color_samples.	Independent-Samples Kruskal-Wallis Test	1,000	Retain the null hypothesis.

10	The distribution of b_day7 is the same across categories of color_samples.	Independent-Samples Kruskal-Wallis Test	,018	Reject the null hypothesis.
11	The distribution of b_day14 is the same across categories of color_samples.	Independent-Samples Kruskal-Wallis Test	,025	Reject the null hypothesis.
12	The distribution of b_day21 is the same across categories of color_samples.	Independent-Samples Kruskal-Wallis Test	,061	Retain the null hypothesis.

a. The significance level is ,050.
b. Asymptotic significance is displayed.

L Day 7

Pairwise Comparisons of color_samples

Sample 1-Sample 2	Test Statistic	Std. Error	Std. Test Statistic	Sig.	Adj. Sig.[a]
NGCV-NGCTCV 75:25	-1,667	4,357	-,383	,702	1,000
NGCV-Uncoated	-2,667	4,357	-,612	,540	1,000
NGCV-NGCTCV 25:75	-6,667	4,357	-1,530	,126	1,000
NGCV-NGCT	9,667	4,357	2,219	,026	,397
NGCV-NGCTCV 50:50	-12,333	4,357	-2,831	,005	,070
NGCTCV 75:25-Uncoated	-1,000	4,357	-,230	,818	1,000
NGCTCV 75:25-NGCTCV 25:75	5,000	4,357	1,148	,251	1,000
NGCTCV 75:25-NGCT	8,000	4,357	1,836	,066	,995

NGCTCV 75:25-NGCTCV 50:50	10,667	4,357	2,448	,014	,215
Uncoated-NGCTCV 25:75	4,000	4,357	,918	,359	1,000
Uncoated-NGCT	7,000	4,357	1,607	,108	1,000
Uncoated-NGCTCV 50:50	9,667	4,357	2,219	,026	,397
NGCTCV 25:75-NGCT	3,000	4,357	,689	,491	1,000
NGCTCV 25:75-NGCTCV 50:50	-5,667	4,357	-1,301	,193	1,000
NGCT-NGCTCV 50:50	-2,667	4,357	-,612	,540	1,000

Each row tests the null hypothesis that the Sample 1 and Sample 2 distributions are the same.

Asymptotic significances (2-sided tests) are displayed. The significance level is ,050.

a. Significance values have been adjusted by the Bonferroni correction for multiple tests.

Day 14

Pairwise Comparisons of color_samples

Sample 1-Sample 2	Test Statistic	Std. Error	Std. Test Statistic	Sig.	Adj. Sig.[a]
NGCV-Uncoated	-3,000	4,359	-,688	,491	1,000
NGCV-NGCTCV 50:50	-6,667	4,359	-1,529	,126	1,000
NGCV-NGCT	8,667	4,359	1,988	,047	,702

NGCV-NGCTCV 75:25	-12,000	4,359	-2,753	,006	,089
NGCV-NGCTCV 25:75	-14,667	4,359	-3,365	<,001	,011
Uncoated-NGCTCV 50:50	3,667	4,359	,841	,400	1,000
Uncoated-NGCT	5,667	4,359	1,300	,194	1,000
Uncoated-NGCTCV 75:25	9,000	4,359	2,065	,039	,584
Uncoated-NGCTCV 25:75	11,667	4,359	2,677	,007	,112
NGCTCV 50:50-NGCT	2,000	4,359	,459	,646	1,000
NGCTCV 50:50-NGCTCV 75:25	-5,333	4,359	-1,224	,221	1,000
NGCTCV 50:50-NGCTCV 25:75	8,000	4,359	1,835	,066	,997
NGCT-NGCTCV 75:25	-3,333	4,359	-,765	,444	1,000
NGCT-NGCTCV 25:75	-6,000	4,359	-1,376	,169	1,000
NGCTCV 75:25-NGCTCV 25:75	2,667	4,359	,612	,541	1,000

Each row tests the null hypothesis that the Sample 1 and Sample 2 distributions are the same.

Asymptotic significances (2-sided tests) are displayed. The significance level is ,050.

a. Significance values have been adjusted by the Bonferroni correction for multiple tests.

L Day 21

Pairwise Comparisons of color_samples

Sample 1-Sample 2	Test Statistic	Std. Error	Std. Test Statistic	Sig.	Adj. Sig.[a]
Uncoated-NGCV	,333	4,359	,076	,939	1,000
Uncoated-NGCT	6,000	4,359	1,376	,169	1,000
Uncoated-NGCTCV 50:50	6,333	4,359	1,453	,146	1,000
Uncoated-NGCTCV 25:75	10,667	4,359	2,447	,014	,216
Uncoated-NGCTCV 75:25	13,667	4,359	3,135	,002	,026
NGCV-NGCT	5,667	4,359	1,300	,194	1,000
NGCV-NGCTCV 50:50	-6,000	4,359	-1,376	,169	1,000
NGCV-NGCTCV 25:75	-10,333	4,359	-2,371	,018	,266
NGCV-NGCTCV 75:25	-13,333	4,359	-3,059	,002	,033
NGCT-NGCTCV 50:50	-,333	4,359	-,076	,939	1,000
NGCT-NGCTCV 25:75	-4,667	4,359	-1,071	,284	1,000
NGCT-NGCTCV 75:25	-7,667	4,359	-1,759	,079	1,000
NGCTCV 50:50-NGCTCV 25:75	4,333	4,359	,994	,320	1,000
NGCTCV 50:50-NGCTCV 75:25	-7,333	4,359	-1,682	,092	1,000

NGCTCV 25:75-NGCTCV 75:25	-3,000	4,359	-,688	,491	1,000

Each row tests the null hypothesis that the Sample 1 and Sample 2 distributions are the same.

Asymptotic significances (2-sided tests) are displayed. The significance level is ,050.

a. Significance values have been adjusted by the Bonferroni correction for multiple tests.

Table S12. Statistical analysis results (Kruskal-Wallis) for L* parameter results

Hypothesis Test Summary				
	**Null Hypothesis**	**Test**	**Sig.**[a,b]	Decision
1	The distribution of L_NGCT is the same across categories of color_day.	Independent-Samples Kruskal-Wallis Test	,016	Reject the null hypothesis.
2	The distribution of L_NGCV is the same across categories of color_day.	Independent-Samples Kruskal-Wallis Test	,016	Reject the null hypothesis.
3	The distribution of L_NGCTCV_25_75 is the same across categories of color_day.	Independent-Samples Kruskal-Wallis Test	,021	Reject the null hypothesis.
4	The distribution of L_NGCTCV_50_50 is the same across categories of color_day.	Independent-Samples Kruskal-Wallis Test	,016	Reject the null hypothesis.
5	The distribution of L_NGCTCV75_25 is the same across categories of color_day.	Independent-Samples Kruskal-Wallis Test	,053	Retain the null hypothesis.
6	The distribution of L_Uncoated is the same across categories of color_day.	Independent-Samples Kruskal-Wallis Test	,016	Reject the null hypothesis.

a. The significance level is ,050.
b. Asymptotic significance is displayed.

a Day 7

Table S13. Statistical analysis results (Kruskal-Wallis) for a* parameter results

Pairwise Comparisons of color_samples

Sample 1-Sample 2	Test Statistic	Std. Error	Std. Test Statistic	Sig.	Adj. Sig.[a]
NGCTCV 50:50-NGCTCV 25:75	1,167	4,357	,268	,789	1,000
NGCTCV 50:50-NGCTCV 75:25	-1,667	4,357	-,383	,702	1,000
NGCTCV 50:50-NGCT	5,167	4,357	1,186	,236	1,000
NGCTCV 50:50-Uncoated	-8,167	4,357	-1,875	,061	,913
NGCTCV 50:50-NGCV	11,833	4,357	2,716	,007	,099
NGCTCV 25:75-NGCTCV 75:25	-,500	4,357	-,115	,909	1,000
NGCTCV 25:75-NGCT	4,000	4,357	,918	,359	1,000
NGCTCV 25:75-Uncoated	-7,000	4,357	-1,607	,108	1,000
NGCTCV 25:75-NGCV	10,667	4,357	2,448	,014	,215
NGCTCV 75:25-NGCT	3,500	4,357	,803	,422	1,000
NGCTCV 75:25-Uncoated	-6,500	4,357	-1,492	,136	1,000
NGCTCV 75:25-NGCV	10,167	4,357	2,334	,020	,294
NGCT-Uncoated	-3,000	4,357	-,689	,491	1,000
NGCT-NGCV	-6,667	4,357	-1,530	,126	1,000

Uncoated-NGCV	3,667	4,357	,842	,400	1,000

Each row tests the null hypothesis that the Sample 1 and Sample 2 distributions are the same.

Asymptotic significances (2-sided tests) are displayed. The significance level is ,050.

a. Significance values have been adjusted by the Bonferroni correction for multiple tests.

a day 14

Pairwise Comparisons of color_samples

Sample 1-Sample 2	Test Statistic	Std. Error	Std. Test Statistic	Sig.	Adj. Sig.[a]
NGCTCV 50:50-NGCTCV 25:75	1,167	4,357	,268	,789	1,000
NGCTCV 50:50-NGCTCV 75:25	-1,667	4,357	-,383	,702	1,000
NGCTCV 50:50-NGCT	5,167	4,357	1,186	,236	1,000
NGCTCV 50:50-Uncoated	-8,167	4,357	-1,875	,061	,913
NGCTCV 50:50-NGCV	11,833	4,357	2,716	,007	,099
NGCTCV 25:75-NGCTCV 75:25	-,500	4,357	-,115	,909	1,000
NGCTCV 25:75-NGCT	4,000	4,357	,918	,359	1,000
NGCTCV 25:75-Uncoated	-7,000	4,357	-1,607	,108	1,000
NGCTCV 25:75-NGCV	10,667	4,357	2,448	,014	,215

NGCTCV 75:25-NGCT	3,500	4,357	,803	,422	1,000
NGCTCV 75:25-Uncoated	-6,500	4,357	-1,492	,136	1,000
NGCTCV 75:25-NGCV	10,167	4,357	2,334	,020	,294
NGCT-Uncoated	-3,000	4,357	-,689	,491	1,000
NGCT-NGCV	-6,667	4,357	-1,530	,126	1,000
Uncoated-NGCV	3,667	4,357	,842	,400	1,000

Each row tests the null hypothesis that the Sample 1 and Sample 2 distributions are the same.

Asymptotic significances (2-sided tests) are displayed. The significance level is ,050.

a. Significance values have been adjusted by the Bonferroni correction for multiple tests.

a Day 21

Pairwise Comparisons of color_samples

Sample 1-Sample 2	Test Statistic	Std. Error	Std. Test Statistic	Sig.	Adj. Sig.[a]
NGCT-Uncoated	-,333	4,359	-,076	,939	1,000
NGCT-NGCTCV 50:50	-5,333	4,359	-1,224	,221	1,000
NGCT-NGCTCV 75:25	-6,667	4,359	-1,529	,126	1,000
NGCT-NGCTCV 25:75	-7,667	4,359	-1,759	,079	1,000
NGCT-NGCV	-13,000	4,359	-2,982	,003	,043
Uncoated-NGCTCV 50:50	5,000	4,359	1,147	,251	1,000

Uncoated-NGCTCV 75:25	6,333	4,359	1,453	,146	1,000
Uncoated-NGCTCV 25:75	7,333	4,359	1,682	,092	1,000
Uncoated-NGCV	12,667	4,359	2,906	,004	,055
NGCTCV 50:50-NGCTCV 75:25	-1,333	4,359	-,306	,760	1,000
NGCTCV 50:50-NGCTCV 25:75	2,333	4,359	,535	,592	1,000
NGCTCV 50:50-NGCV	7,667	4,359	1,759	,079	1,000
NGCTCV 75:25-NGCTCV 25:75	1,000	4,359	,229	,819	1,000
NGCTCV 75:25-NGCV	6,333	4,359	1,453	,146	1,000
NGCTCV 25:75-NGCV	5,333	4,359	1,224	,221	1,000

Each row tests the null hypothesis that the Sample 1 and Sample 2 distributions are the same.

Asymptotic significances (2-sided tests) are displayed. The significance level is ,050.

a. Significance values have been adjusted by the Bonferroni correction for multiple tests.

Table S14. Statistical analysis results (Kruskal-Wallis) for a* parameter results

Hypothesis Test Summary

	Null Hypothesis	Test	Sig.[a,b]	Decision
1	The distribution of a_NGCT is the same across categories of color_day.	Independent-Samples Kruskal-Wallis Test	,034	Reject the null hypothesis.

2	The distribution of a_NGCV is the same across categories of color_day.	Independent-Samples Kruskal-Wallis Test	,025	Reject the null hypothesis.
3	The distribution of a_NGCTCV_25_75 is the same across categories of color_day.	Independent-Samples Kruskal-Wallis Test	,038	Reject the null hypothesis.
4	The distribution of a_NGCTCV_50_50 is the same across categories of color_day.	Independent-Samples Kruskal-Wallis Test	,052	Retain the null hypothesis.
5	The distribution of a_NGCTCV_75_25 is the same across categories of color_day.	Independent-Samples Kruskal-Wallis Test	,121	Retain the null hypothesis.
6	The distribution of a_Uncoated is the same across categories of color_day.	Independent-Samples Kruskal-Wallis Test	,055	Retain the null hypothesis.

a. The significance level is ,050.

b. Asymptotic significance is displayed.

b Day 7

Table S15. Statistical analysis results (Kruskal-Wallis) for b* parameter results

Pairwise Comparisons of color_samples

Sample 1-Sample 2	Test Statistic	Std. Error	Std. Test Statistic	Sig.	Adj. Sig.[a]

NGCTCV 25:75-NGCTCV 75:25	-,167	4,354	-,038	,969	1,000
NGCTCV 25:75-NGCV	,333	4,354	,077	,939	1,000
NGCTCV 25:75-NGCT	7,333	4,354	1,684	,092	1,000
NGCTCV 25:75-NGCTCV 50:50	-8,833	4,354	-2,029	,042	,637
NGCTCV 25:75-Uncoated	-11,333	4,354	-2,603	,009	,139
NGCTCV 75:25-NGCV	,167	4,354	,038	,969	1,000
NGCTCV 75:25-NGCT	7,167	4,354	1,646	,100	1,000
NGCTCV 75:25-NGCTCV 50:50	8,667	4,354	1,990	,047	,698
NGCTCV 75:25-Uncoated	-11,167	4,354	-2,564	,010	,155
NGCV-NGCT	7,000	4,354	1,608	,108	1,000
NGCV-NGCTCV 50:50	-8,500	4,354	-1,952	,051	,764
NGCV-Uncoated	-11,000	4,354	-2,526	,012	,173
NGCT-NGCTCV 50:50	-1,500	4,354	-,344	,730	1,000
NGCT-Uncoated	-4,000	4,354	-,919	,358	1,000
NGCTCV 50:50-Uncoated	-2,500	4,354	-,574	,566	1,000

Each row tests the null hypothesis that the Sample 1 and Sample 2 distributions are the same.

Asymptotic significances (2-sided tests) are displayed. The significance level is ,050.

a. Significance values have been adjusted by the Bonferroni correction for multiple tests.

b Day 14

Pairwise Comparisons of color_samples

Sample 1-Sample 2	Test Statistic	Std. Error	Std. Test Statistic	Sig.	Adj. Sig.[a]
NGCV-Uncoated	-1,000	4,359	-,229	,819	1,000
NGCV-NGCTCV 50:50	-6,667	4,359	-1,529	,126	1,000
NGCV-NGCT	9,667	4,359	2,218	,027	,399
NGCV-NGCTCV 25:75	-10,000	4,359	-2,294	,022	,327
NGCV-NGCTCV 75:25	-11,667	4,359	-2,677	,007	,112
Uncoated-NGCTCV 50:50	5,667	4,359	1,300	,194	1,000
Uncoated-NGCT	8,667	4,359	1,988	,047	,702
Uncoated-NGCTCV 25:75	9,000	4,359	2,065	,039	,584
Uncoated-NGCTCV 75:25	10,667	4,359	2,447	,014	,216
NGCTCV 50:50-NGCT	3,000	4,359	,688	,491	1,000
NGCTCV 50:50-NGCTCV 25:75	3,333	4,359	,765	,444	1,000
NGCTCV 50:50-NGCTCV 75:25	-5,000	4,359	-1,147	,251	1,000
NGCT-NGCTCV 25:75	-,333	4,359	-,076	,939	1,000
NGCT-NGCTCV 75:25	-2,000	4,359	-,459	,646	1,000

NGCTCV 25:75-NGCTCV 75:25	-1,667	4,359	-,382	,702	1,000

Each row tests the null hypothesis that the Sample 1 and Sample 2 distributions are the same.

Asymptotic significances (2-sided tests) are displayed. The significance level is ,050.

a. Significance values have been adjusted by the Bonferroni correction for multiple tests.

b_ Day 21

Pairwise Comparisons of color_samples

Sample 1-Sample 2	Test Statistic	Std. Error	Std. Test Statistic	Sig.	Adj. Sig.[a]
NGCV-NGCTCV 75:25	-4,667	4,359	-1,071	,284	1,000
NGCV-NGCTCV 50:50	-7,667	4,359	-1,759	,079	1,000
NGCV-Uncoated	-10,000	4,359	-2,294	,022	,327
NGCV-NGCTCV 25:75	-11,000	4,359	-2,524	,012	,174
NGCV-NGCT	11,667	4,359	2,677	,007	,112
NGCTCV 75:25-NGCTCV 50:50	3,000	4,359	,688	,491	1,000
NGCTCV 75:25-Uncoated	-5,333	4,359	-1,224	,221	1,000
NGCTCV 75:25-NGCTCV 25:75	6,333	4,359	1,453	,146	1,000
NGCTCV 75:25-NGCT	7,000	4,359	1,606	,108	1,000

NGCTCV 50:50-Uncoated	-2,333	4,359	-,535	,592	1,000
NGCTCV 50:50-NGCTCV 25:75	3,333	4,359	,765	,444	1,000
NGCTCV 50:50-NGCT	4,000	4,359	,918	,359	1,000
Uncoated-NGCTCV 25:75	1,000	4,359	,229	,819	1,000
Uncoated-NGCT	1,667	4,359	,382	,702	1,000
NGCTCV 25:75-NGCT	,667	4,359	,153	,878	1,000

Each row tests the null hypothesis that the Sample 1 and Sample 2 distributions are the same.

Asymptotic significances (2-sided tests) are displayed. The significance level is ,050.

a. Significance values have been adjusted by the Bonferroni correction for multiple tests.

Table S16. Statistical analysis results (Kruskal-Wallis) for b* parameter results

Hypothesis Test Summary

	Null Hypothesis	Test	Sig.[a,b]	Decision
1	The distribution of b_NGCT is the same across categories of color_day.	Independent-Samples Kruskal-Wallis Test	,057	Retain the null hypothesis.
2	The distribution of b_NGCV is the same across categories of color_day.	Independent-Samples Kruskal-Wallis Test	,016	Reject the null hypothesis.
3	The distribution of b_NGCTCV_25_75 is the same across categories of color_day.	Independent-Samples Kruskal-Wallis Test	,025	Reject the null hypothesis.

4	The distribution of b_NGCTCV_50_50 is the same across categories of color_day.	Independent-Samples Kruskal-Wallis Test	,024	Reject the null hypothesis.
5	The distribution of b_NGCTCV_75_25 is the same across categories of color_day.	Independent-Samples Kruskal-Wallis Test	,022	Reject the null hypothesis.
6	The distribution of b_Uncoated is the same across categories of color_day.	Independent-Samples Kruskal-Wallis Test	,016	Reject the null hypothesis.

a. The significance level is ,050.

b. Asymptotic significance is displayed.

Table S17. Statistical analysis results (Kruskal-Wallis) for sensory analysis results

Hypothesis Test Summary

	Null Hypothesis	Test	Sig.[a,b]	Decision
1	The distribution of Results is the same across categories of Treatment.	Independent-Samples Kruskal-Wallis Test	<,001	Reject the null hypothesis.
2	The distribution of Results_odor is the same across categories of Treatment.	Independent-Samples Kruskal-Wallis Test	<,001	Reject the null hypothesis.
3	The distribution of Results_color is the same across categories of Treatment.	Independent-Samples Kruskal-Wallis Test	<,001	Reject the null hypothesis.
4	The distribution of Results_texture is the same across categories of Treatment.	Independent-Samples Kruskal-Wallis Test	<,001	Reject the null hypothesis.

a. The significance level is ,050.

b. Asymptotic significance is displayed.

Table S18. Statistical analysis results (Kruskal-Wallis) for sensory analysis results

Hypothesis Test Summary

	Null Hypothesis	Test	Sig.[a,b]	Decision
1	The distribution of taste_day_0 is the same across categories of Treatments_sensory.	Independent-Samples Kruskal-Wallis Test	1,000	Retain the null hypothesis.

2	The distribution of taste_day_7 is the same across categories of Treatments_sensory.	Independent-Samples Kruskal-Wallis Test	<,001	Reject the null hypothesis.
3	The distribution of taste_day_14 is the same across categories of Treatments_sensory.	Independent-Samples Kruskal-Wallis Test	<,001	Reject the null hypothesis.
4	The distribution of taste_day_21 is the same across categories of Treatments_sensory.	Independent-Samples Kruskal-Wallis Test	<,001	Reject the null hypothesis.
5	The distribution of odor_day_0 is the same across categories of Treatments_sensory.	Independent-Samples Kruskal-Wallis Test	1,000	Retain the null hypothesis.
6	The distribution of odor_day_7 is the same across categories of Treatments_sensory.	Independent-Samples Kruskal-Wallis Test	<,001	Reject the null hypothesis.
7	The distribution of odor_day_14 is the same across categories of Treatments_sensory.	Independent-Samples Kruskal-Wallis Test	<,001	Reject the null hypothesis.
8	The distribution of odor_day_21 is the same across categories of Treatments_sensory.	Independent-Samples Kruskal-Wallis Test	<,001	Reject the null hypothesis.
9	The distribution of color_day_0 is the same across categories of Treatments_sensory.	Independent-Samples Kruskal-Wallis Test	1,000	Retain the null hypothesis.
10	The distribution of color_day_7 is the same across categories of Treatments_sensory.	Independent-Samples Kruskal-Wallis Test	<,001	Reject the null hypothesis.

11	The distribution of color_day_14 is the same across categories of Treatments_sensory.	Independent-Samples Kruskal-Wallis Test	<,001	Reject the null hypothesis.
12	The distribution of color_day_21 is the same across categories of Treatments_sensory.	Independent-Samples Kruskal-Wallis Test	<,001	Reject the null hypothesis.
13	The distribution of texture_day_0 is the same across categories of Treatments_sensory.	Independent-Samples Kruskal-Wallis Test	1,000	Retain the null hypothesis.
14	The distribution of texture_day_7 is the same across categories of Treatments_sensory.	Independent-Samples Kruskal-Wallis Test	<,001	Reject the null hypothesis.
15	The distribution of texture_day_14 is the same across categories of Treatments_sensory.	Independent-Samples Kruskal-Wallis Test	<,001	Reject the null hypothesis.
16	The distribution of texture_day_21 is the same across categories of Treatments_sensory.	Independent-Samples Kruskal-Wallis Test	<,001	Reject the null hypothesis.

a. The significance level is ,050.

b. Asymptotic significance is displayed.
